# NTSR1 glycosylation and MMP dependent cleavage generate three distinct forms of the protein

**DOI:** 10.1038/s41598-023-31790-7

**Published:** 2023-03-22

**Authors:** Fotine Libanje, Raphael Delille, Pamela A Young, Sylvie Rolland, Florence Meyer-Losic, Elodie Lewkowicz, Stephan Klinz

**Affiliations:** 1 Translational Biomarkers and Pharmacology, IPSEN Innovation, Les Ulis, France; 2 Early Development and Translational Sciences, IPSEN Bioscience, Cambridge, USA

**Keywords:** Biochemistry, Biological techniques, Cancer, Cell biology, Molecular biology

## Abstract

NTSR1 abnormal expression by cancer cells makes it a strategic target for antitumoral therapies, such as compounds that use NTSR1 binding probes to deliver cytotoxic agents to tumor cells. Success of these therapies relies on NTSR1 protein availability and accessibility; therefore, understanding the protein’s biology is crucial. We studied NTSR1 protein in exogenously and endogenously expressing non-tumoral and tumoral cells. We found NTSR1 to be expressed as three distinct protein forms: the NTSR1-high form, a glycosylated protein; the NTSR1-low form, a N-terminally cleaved and de-glycosylated protein; and the NTSR1-LP protein with the MW size predicted by its NTSR1 amino acid sequence. We show that the NTSR1-high form is cleaved by MMPs to generate the NTSR1-low form, a process that is promoted by the Neurotensin (NTS) ligand. In addition, NTS induced the internalization of plasma membrane localized NTSR1 and degradation of NTSR1-low form via the proteasome. Importantly, we found NTSR1-low form to be the most abundant form in the tumoral cells and in PDAC Patient Derived Xenograft, demonstrating its physiopathological relevance. Altogether, our work provides important technical and experimental tools as well as new crucial insights into NTSR1 protein biology that are required to develop clinically relevant NTSR1 targeting anti-tumoral therapies.

## Introduction

Neurotensin receptor 1 (NTSR1) is a receptor for neurotensin (NTS), along with NTSR2 and NTSR3/Sortilin^1^. Both NTSR1 and NTSR2 are physiologically expressed in the adult central and peripheral nervous system^2,3^ and are rhodopsin-like class A G protein coupled receptor (GPCR)^4^, a family of protein consisting of 7 transmembrane domains with extracellular N-terminal and intracellular C-terminal tails. NTSR3/Sortilin, a single transmembrane domain Type I receptor^5^, which like NTSR1 is a high affinity NTS receptor, is however structurally unrelated to NTSR1 and NTSR2^6,7^.

NTSR1 expression and activation by NTS have been associated with several cancers including colorectal cancer^8^ and pancreatic cancer^9–15^ making the protein an interesting target for antitumoral therapy. After clinical failure of the NTSR1-antagonizing molecule, Meclinertant^9,16,17^, new NTSR1-targeting antitumoral strategies have emerged with compounds that deliver cytotoxic agents to NTSR1-expressing tumoral cells via NTSR1-binding domains^18–21^. As the efficacy of these drugs relies on the abundance and the accessibility of NTSR1, understanding NTSR1 protein expression, stability, trafficking and post-translational modification (PTM) is crucial.

Until now, due to the lack of in vitro tools including validated anti-NTSR1 antibodies^22^, NTSR1 protein expression was mainly assessed by gene expression, downstream pathway signaling activity or NTS binding^8,9,11,23,24^. These experimental approaches do not allow the protein’s PTM study and limit the biological understanding of the protein itself as gene expression data do not always correlate to protein expression, as signaling activity provides only limited information on the protein biology, and as NTS binding assays do not discriminate between the different NTS receptors.

At the amino sequence level, NTSR1 possess three putative N-glycosylation sites Asn-X-Ser (X ≠ Pro), all located on the N-terminal ectodomain of the protein. Glycosylation is a PTM which links glycan motifs to the proteins^25^. N-glycosylation is the most prevalent and occurs co-translationally. It is initiated in the endoplasmic reticulum and fully elongated in the Golgi. N-glycosylation has been previously shown to be important for GPCRs folding, export from ER to Golgi and transport to the plasma membrane^25,26^. O-glycosylation, which is initiated in the Golgi, is a type of glycosylation that happens on a Serine or a Threonine residue^25^. Until now, despite the presence of the putative N-glycosylation site, NTSR1 glycosylation status remains unknown along with the receptor’s PTMs.

In our study, in addition to identifying and validating experimental tools, we used in vitro non tumoral and physiopathologically relevant colorectal (CRC) and pancreatic ductal adenocarcinoma (PDAC) cancer cell models to study NTSR1 biology.

## Results

### NTSR1 is expressed as three protein forms

**Figure 1:**
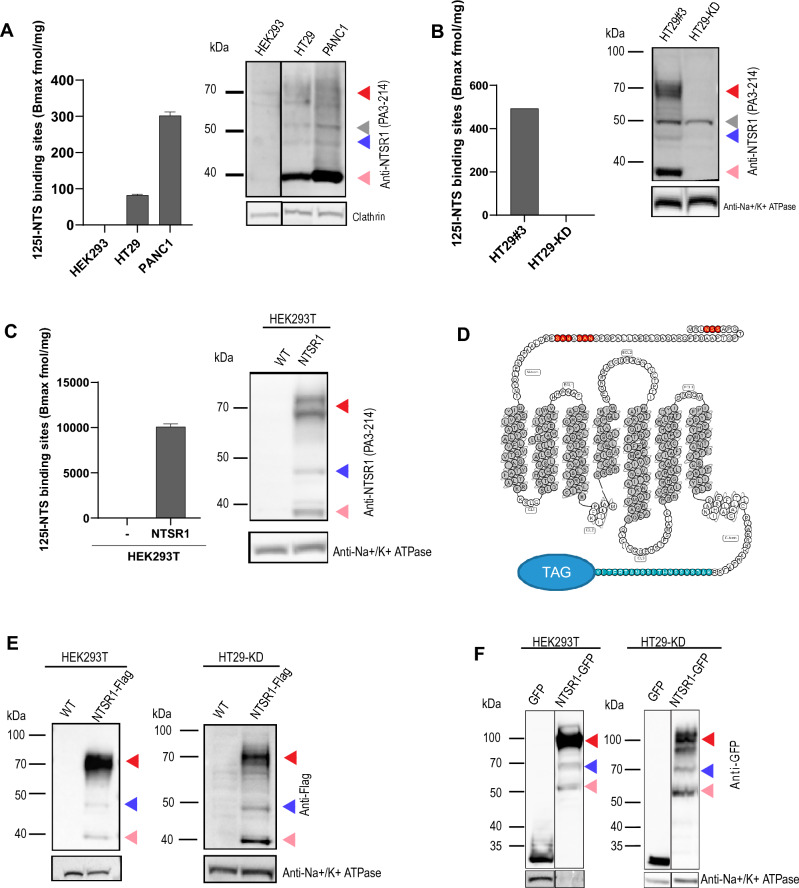
NTSR1 is expressed as three protein forms of distinct MW. **(A–F**) Arrowheads: Red: NTSR1-high, pink: NTSR1-low, blue: NTSR1-LP, grey: nonspecific band. (**A**) Left, ^125^I-NTS binding assay NTS Bmax values measured on HEK293 WT, HT29 and PANC1 total cell membranes, + /- SEM, N = 2. Right, representative anti-NTSR1 WB performed after SDS-PAGE of total GPCR fraction extracted from total membranes of HEK WT, HT29 and PANC1 cells. Clathrin is the loading control. (**B**) Left, ^125^I-NTS binding assay NTS Bmax values measured on HT29-KD (NTSR1-depleted by CRIPSR-cas9) or HT29#3 (HT29 clone). Right, representative anti-NTSR1 WB performed after SDS-PAGE of total GPCR fraction extracted from total membranes of HT29-KD (NTSR1-depleted by CRIPSR-cas9) or HT29#3 (HT29 isolated clone); Na + /K + ATPase is the loading control. (**C**) Left, ^125^I-NTS binding assay mean NTS Bmax values measured on HEK293T overexpressing NTSR1 or not, + /- SEM, N = 2. Right, representative anti-NTSR1 WB performed after SDS-PAGE of SDS lysates from HEK293T cells overexpressing NTSR1 or not (WT). NTSR1 (PA3-214 antibody) is immunoblotted and Na + /K + ATPase is the loading control. (**D**) Snake diagram of NTSR1’s amino-acid sequence. The 3 predicted glycosylation sites are displayed in red, the epitope of the anti-NTSR1 antibody PA3-214 is displayed in blue and the position of the eventual C-terminal tags (GFP or FLAG) is highlighted by “TAG”. The transmembrane domains are filled in gray. The diagram was created with GPCRdb server (http://www.gpcrdb.org). (**E**) Representative anti-FLAG WB performed after SDS-PAGE of total GPCR fraction extracted from the total cell lysate of HEK293T or HT29-KD overexpressing NTSR1 tagged with FLAG at its C-terminus (NTSR1-flag) or not (WT). Na + /K + ATPase is the loading control. (**F**) Representative anti-GFP WB performed after SDS-PAGE of total GPCR fraction extracted from the total cell lysate of HEK293T or HT29-KD overexpressing GFP alone (GFP) or NTSR1 tagged with GFP at its C-terminus (NTSR-GFP). Na + /K + ATPase is the loading control. Uncropped WB blots images are included in Supplementary information.

Historically, NTSR1 expression in HT29 and PANC1 was mainly assessed through the ^125^I-NTS binding assay^7,12,27^. When performed on total cell membranes (plasma and cytosolic membranes), this assay measures total NTS binding sites (Bmax, fmol/mg), which corresponds to NTS receptor abundance. To confirm previous results and validate NTS receptor expression in our HT29 and PANC1 cell lines, we performed ^125^I-NTS binding assay using HEK293, a non-tumoral cell line devoid of Neurotensin receptors, as a negative control^28^. Whereas no ^125^I-NTS binding was detected in HEK293 membranes, a Bmax of 82 fmol/mg for HT29 and 302 fmol/mg for PANC1 was measured, confirming that PANC1 and HT29 express NTS receptors as previously described (Fig. 1A).

Because the ^125^I-NTS binding assay does not allow the distinction between the expression of NTSR1 and NTSR3, the two high affinity NTS receptors^1,29^, we aimed at directly detecting NTSR1 expression in PANC1 and HT29 cells by Western Blot (WB) analysis (Fig. 1A). Most commercially available anti-NTSR1 antibodies yielded multiple bands in WB, therefore, to reduce the non-specific signal, we isolated total cell membranes and extracted the GPCR fraction for SDS/PAGE (suppl. Fig. 1). Using siRNA and shRNA, we were able to discriminate between non-specific (50 kDa) and NTSR1 specific bands (suppl. Fig. 2).

We further generated a negative control, using CrispR-Cas9 to knock down NTSR1 (sgNTSR1) (suppl. Fig. 2), and isolated the NTSR1-depleted HT29 clone (HT29-KD).^125^I-NTS binding assay revealed the complete loss of NTS binding of this clone (Fig. 1B). Accordingly, SDS-PAGE/WB confirmed the complete loss of NTSR1 specific bands while the non-specific band around the 50 kDa MW size was maintained (Fig. 1B). These results demonstrate that our HT29 and PANC1 cell lines express NTSR1 and that NTSR1 but not NTSR3 is responsible for NTS binding to HT29 cells.

Surprisingly, NTSR1 specific signal consisted of three distinct molecular weight bands: a band with an apparent size of 46 kDa that correlates with NTSR1 protein predicted size based on its amino acid sequence (referred to as NTSR1-less processed or NTSR1-LP), a low MW band that migrated below 40 kDa (referred to as NTSR1-low) and a high MW band detected around 70 kDa (referred to as NTSR1-high) (Fig. 1B). Through clonal selection, we identified the clone HT29#3 (Bmax = 493 fmol/mg) that expressed NTSR1 levels 6 times higher than the parental cell population (HT29 Bmax 82 fmol/mg), which allowed a stronger detection of the three NTSR1 species (Fig. 1B, suppl. Fig. 2). In conclusion, our results confirm that our HT29 and PANC1 cell lines express NTSR1, and further demonstrate the existence of three different NTSR1 protein forms.

To further characterize these three NTSR1 protein forms, we asked whether they could be NTSR1 isoforms or splicing variants. We stably overexpressed a non-tagged form of NTSR1 by transduction of a NTSR1 cDNA in HEK293T cells which do not express NTSR1, which eliminates the event of protein isoform expression or mRNA splicing variation (Fig. 1C). ^125^I-NTS binding assay again demonstrated the ability of the overexpressed NTSR1 protein to bind NTS. The high Bmax value (10 pmol/mg) reflects high NTSR1 expression levels (20-fold higher) compared to HT29 and PANC1 (Fig. 1C). In line with NTSR1 expression observed in PANC1 and HT29 cells, SDS-PAGE/WB analysis of the HEK293T-NTSR1 also revealed three distinct WB bands specific to NTSR1 protein with the same apparent molecular weight of 46 kDa, below 40 kDa and 70 kDa (Fig. 1C). The translation of NTSR1 cDNA into the three NTSR1 forms demonstrates that these NTSR1 forms are not protein isoforms or splicing variants.

To exclude any experimental bias brought by the anti-NTSR1 antibody targeting the C-terminal extremity of the protein, we generated C-terminally GFP-tagged (HEK293T-NTSR1GFP) or FLAG-tagged NTSR1 (HEK293T-NTSR1FLAG)-overexpressing HEK293T and performed WB, using antibodies either targeting the FLAG or the GFP tag (Fig. 1D,E,F, suppl. Fig. 3). As identified with the anti-NTSR1 antibody, anti-GFP and anti-FLAG antibodies targeting NTSR1-FLAG or NTSR1-GFP also detected the three NTSR1 molecular weight species (Fig. 1D,E,F). As expected, the NTSR1-FLAG protein species resolved at the same apparent molecular weight as the non-tagged NTSR1 whereas the NTSR1-GFP species had a mobility shift of around 27 kDa corresponding to GFP molecular weight. Altogether, we have shown that three forms of NTSR1 protein, NTSR1-high, NTSR1-low and NTSR1-LP, are co-expressed and do not result from NTSR1 experimental bias, isoform expression nor from splicing variant but may result from PTM of the protein.

### Neurotensin regulates the relative abundance and stability of the NTSR1 protein forms

Before investigating the PTM nature of the three NTSR1 protein forms, we verified their biological relevance by testing the impact of the NTSR1 natural ligand, NTS, on their expression.

**Figure 2:**
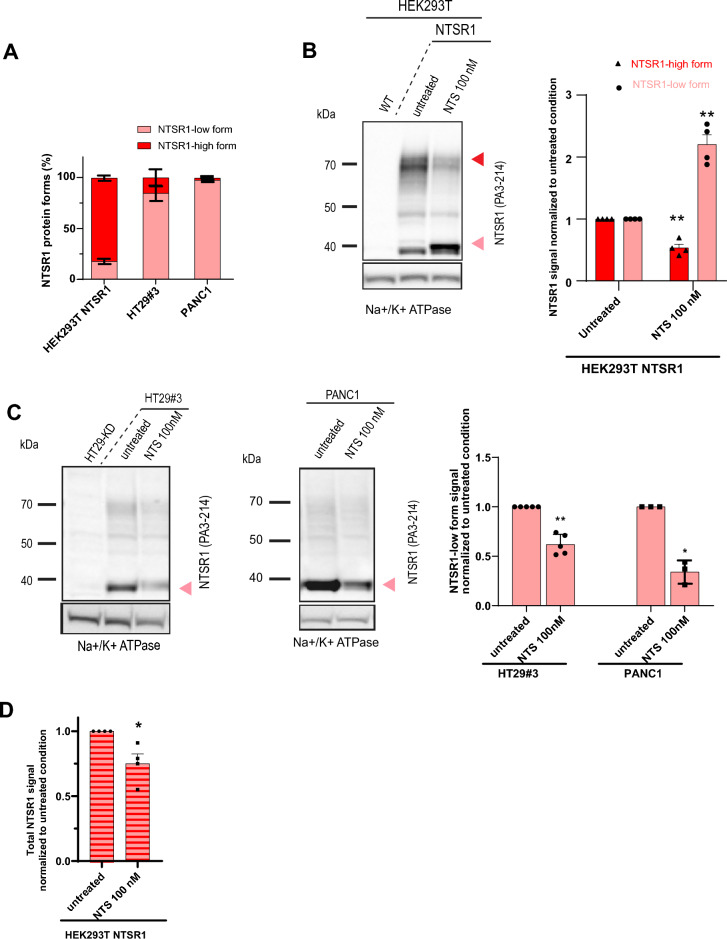
Neurotensin regulates the relative abundance and stability of NTSR1 protein forms. (**A–C**) Arrowheads: Red: NTSR1-high, pink: NTSR1-low. (**A**) Percentage of NTSR1-high and NTSR1-low protein forms detected by anti-NTSR1 WB in the cell lines, + /-SEM, N = 3. (**B**) Left, Representative anti-NTSR1 WB performed after SDS-PAGE of SDS total cell lysate of HEK293T cells overexpressing NTSR1 (NTSR1) or not (WT). HEK293T-NTSR1 were treated or not (untreated) with 100 nM of Neurotensin for 1 h (NTS 100 nM). Na + /K + ATPase is used as the loading control. Right, Anti-NTSR1 WB quantification of NTSR1-low and NTSR1-high protein forms as presented in B, left. The NTSR1 signal is normalized to Na + /K + ATPase signal, before normalization to the untreated condition, + /- SEM, N = 4. (**C**) Left, representative anti-NTSR1 WB performed after SDS-PAGE of total GPCR fraction extracted from total membranes of HT29-KD (NTSR1 depleted), HT29#3 (HT29 clone) and PANC1 cells. Cells were treated or not (untreated) with 100 nM of Neurotensin for 1 h (NTS 100 nM). HT29-KD are used as negative control. Na + /K + ATPase is used as the loading control. Right, Anti-NTSR1 WB quantification of NTSR1-low and NTSR1-high protein forms. The NTSR1 signal is normalized to Na + /K + ATPase signal, before normalization to the untreated condition. + /- SEM, HT29#3, N = 4, PANC1, N = 3, t-test. (**D**) Right Anti-NTSR1 WB quantification of total NTSR1 protein forms (NTSR1-high, -low and -LP) in HEK293T-NTSR1 treated or not (untreated) with 100 nM of Neurotensin for 1 h (NTS 100 nM) as presented in A. The NTSR1 signal is normalized to Na + /K + ATPase signal, before normalization to the untreated condition, + /- SEM, N = 4, t-test. *p < 0.05; **p < 0.01. Uncropped WB blots images are included in Supplementary information.

Even though the three NTSR1 protein forms were detected in the endogenously expressing cancer cells, PANC1 and HT29, as well as in HEK293T-NTSR1 cells overexpressing the protein, the relative abundance of the three NTSR1 forms was not equivalent. The NSTR1-high form was predominant in HEK293T-NTSR1 cells whereas the NTSR1-low form was the most detected in HT29 and PANC1 cells (Fig. 2A). We therefore investigated expression of the NTSR1-high form in HEK293T-NTSR1 cells and NTSR1-low form in the HT29 and PANC1 cell lines (Fig. 2B,C). The cells were treated with 100 nM NTS for 1 h before being subjected to SDS-PAGE/WB (Fig. 2B, suppl. Fig. 4). Intriguingly, NTS treatment induced a decrease of the NTSR1-high form and a concomitant increase of the NTSR1-low form in HEK293T-NTSR1 cells (Fig. 2B). This concomitant and inverse fluctuation of the low and high forms of NTSR1 protein suggests that NTS promotes an exchange from the NTSR1-high form to the NTSR1-low form. To monitor the fate of NTSR1-low form independently of the NTSR1-high form switch, we used PANC1 and HT29#3 cells. After treatment with 100 nM NTS, cells underwent SDS-PAGE/WB analysis (Fig. 2C). Contrary to the observation made in HEK293T-NTSR1 cells, the NTSR1-low form decreased after NTS treatment suggesting that NTS promotes NTSR1-low form degradation. Accordingly, a decrease of the total NTSR1 protein was measured in HEK293T-NTSR1 cells (Fig. 2D) further supporting NTS-induced NTSR1 degradation occurring via NTSR1-low form degradation. These results prompted us to hypothesize that NTS promotes NTSR1-high form transformation into the NTSR1-low form, as observed in HEK293T-NTSR1 cells, and the subsequent NTSR1-low form degradation that is observed in HT29 and PANC1 cells. Altogether, these results indicate that NTSR1 protein forms are biologically relevant and that NTS regulates their expression and stability.

### N-glycosylation is responsible for NTSR1-high protein form

**Figure 3:**
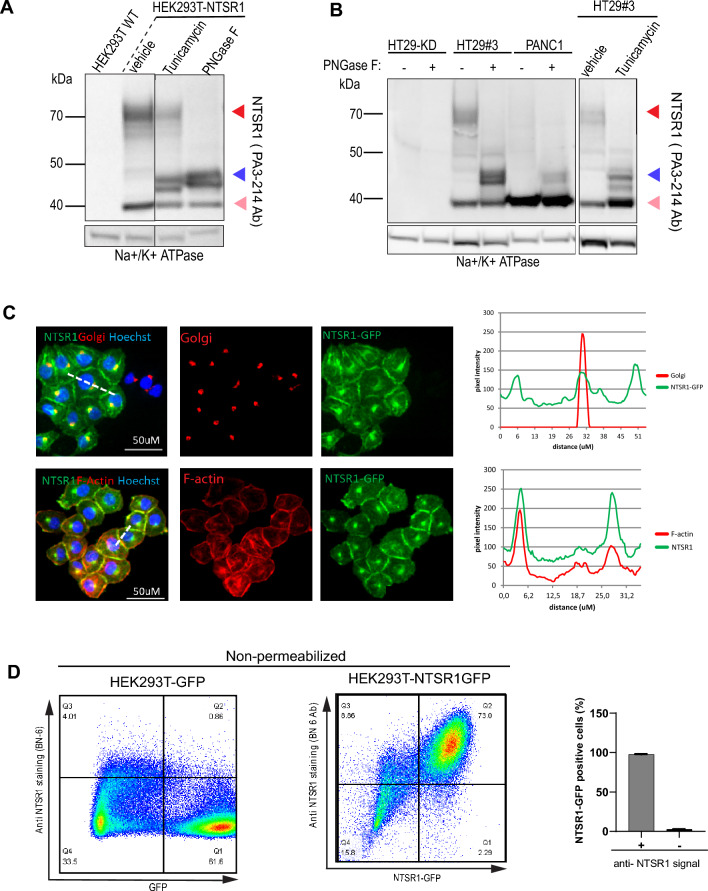
NTSR1 Glycosylation through the secretory pathway generates the proteins higher MW form. (**A**, **B**) Arrowheads: Red: NTSR1-high, pink: NTSR1-low, blue: NTSR1-LP. (**A**) Anti-NTSR1 WB performed on cell lysate from HEK293T cells overexpressing NTSR1 or not (WT). Cells were treated or not (untreated) with DMSO (vehicle) or with 2 µg/mL of the N-glycosylation inhibitor Tunicamycin for 24 h (tunicamycin) or untreated cell lysate was digested with the N-Glycosylase PNGase F (PNase F). Na + /K + ATPase is used as the loading control. (**B**) Representative anti-NTSR1 WB performed after SDS-PAGE of total GPCR fraction extracted from total membranes of HT29-KD (NTSR1-depleted by CRIPSR-cas9) or HT29#3 (HT29 isolated clone) were treated or not (untreated) with DMSO (vehicle) or with 2 µg/mL of the N-glycosylation inhibitor Tunicamycin for 24 h (tunicamycin) or untreated cell lysate was digested with the N-Glycosylase PNGase F (PNase F). HT29-KD is used as a negative control. Na + /K + ATPase is used as the loading control. (**C**) Representative confocal images of PANC1 cells overexpressing the C-terminal GFP-tagged NTSR1 protein. Cells were fixed and stained with anti-GM130 (Golgi), with Phalloidin (F-actin) and with Hoechst. GFP, Phalloidin and Golgi intensity were measured along the line scans applied on the images (dotted lines), and the pixels intensities values are presented. (**D**) FACS analysis of non-permeabilized HEK293T cells overexpressing the C-terminal GFP-tagged NTSR1 (NTSR1 GFP) construct or the GFP alone immunostained with an anti-NTSR1 antibody (BN-6). Right, quantification of GFP positive cells and anti-NTSR1 positive cells, + /- SEM, N = 3. Uncropped WB blots images are included in Supplementary information.

We then sought to determine the PTM responsible for the NTSR1-high form. Because the NTSR1-high form was resistant to strong denaturing conditions and agents (suppl. Fig. 5), we excluded the possibility of the NTSR1-high form to be a dimer or polymer form of the protein^30–33^. As most GPCRs are known to be glycosylated^25,34^ and as NTSR1 contains three putative N-glycosylation sites in its N-terminal end (Fig. 1D), we tested whether NTSR1-high form was a glycosylated protein. We treated HEK293T-NTSR1 cells with Tunicamycin, an inhibitor of N-Glycosylation, before SDS-PAGE/WB analysis (Fig. 3A). Tunicamycin induced a decrease of the NTSR1-high 70 kDa WB band while inducing an increase of the 46 kDa band corresponding to NTSR1-LP form, showing that the NTSR1-high form is a product of NTSR1-LP form glycosylation. As Tunicamycin only affects newly translated proteins, NTSR1-high proteins formed prior to the treatment could still be detected (Fig. 3A). To further demonstrate NTSR1-high glycosylation status, we treated HEK293T-NTSR1, HEK293T-NTSR1-GFP and NTSR1-FLAG cell lysates with PNGase-F, a deglycosylase enzyme that removes N-Glycan backbones from all proteins, and performed SDS-PAGE/ WB assays (Fig. 3A; suppl. Fig. 6C). PNGase-F treatment resulted in negligible detection of the NTSR1-high form, while an increase of the NTSR1-LP form was observed, confirming the results obtained with Tunicamycin. To assess the physiological relevance of our findings we treated HT29 and PANC1 cancer cells with Tunicamycin and PNGase-F (Fig. 3B) which, as in HEK293T-NTSR1 cells, induced a strong increase of the NTSR1-LP form, while the NTSR1-high form was undetectable. All these results demonstrate that NTSR1 is N-glycosylated to form the NTSR1-high form.

To investigate NTSR1 O-glycosylation, we treated HEK293T-NTSR1 and HT29 cells with the O-glycosylation competitive inhibitor BADG (benzyl-2-acetoamino-2-deoxy-α-D-galactopyranoside) which decreased the expression levels of the NTSR1-high form and induced the appearance of multiple lower bands that suggest different states of interim protein N-glycosylation elongation (suppl. Fig. 6D,E). This unexpected results may be due to the previously described BADG induced competitive inhibition of the cells’ whole glycosylation machinery^35,36^, which here disrupts NTSR1 N-glycosylation elongation that normally occurs in the Golgi.

We then investigated NTSR1 localization in PANC1 cells that overexpressed NTSR1-GFP and found that NTSR1 colocalized with the Golgi marker GM130 (Fig. 3C). GPCR biosynthesis usually occurs through the secretory pathway that comprises glycosylation initiation in the endoplasmic reticulum, glycosylation maturation in the Golgi, and trafficking to the plasma membrane. As our results demonstrate that NTSR1 traffics through the ER and the Golgi, we verified NTSR1 plasma membrane localization by staining for F-actin, that is found enriched at the cell periphery. We found colocalization of NTSR1 with the peripheral F-actin, strongly suggesting a plasma membrane localization of the receptor (Fig. 3C). To further confirm NTSR1 protein delivery to the plasma membrane, we used non-permeabilized HEK293T-NTSR1-GFP cells to detect NTSR1 protein by FACS immunostaining. The absence of membrane permeabilization allows the detection of only NTSR1 proteins localized at the plasma membrane (Fig. 3D). HEK293T cells overexpressing GFP alone, used as negative controls, were positive for GFP signal but had no positive anti-NTSR1 signal. By comparison, all HEK293T-NTSR1GFP cells were GFP positive and were immunostained with the anti-NTSR1 antibody confirming plasma membrane localization of NTSR1.

Thus, we show that NTSR1 protein is glycosylated and processed to form the NTSR1-high form. This may occur through the secretory pathway, which delivers the protein to the plasma membrane. Of note, the NTSR1-low form was not affected by the glycosylation-destabilizing treatments indicating that NTSR1-low form does not carry any glycosylation modification (Fig. 3A,B, suppl. Fig. 6A,B).

### NTSR1-high is N-terminally cleaved by matrix metalloproteinase to form the NTSR1-low protein form

**Figure 4:**
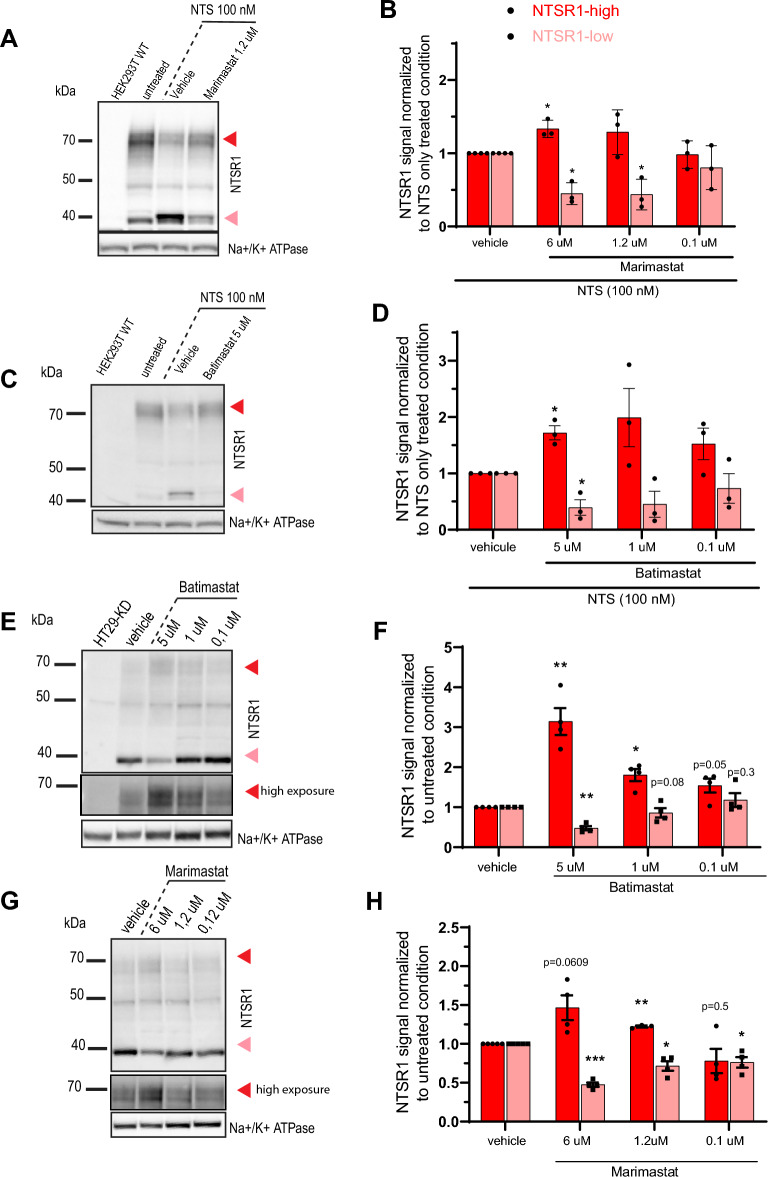
NTSR1-high form is N-terminally cleaved by matrix metalloproteinase to form the NTSR1-low protein form. (**A**, **C**, **E**, **G**) Arrowheads: Red: NTSR1-high, pink: NTSR1-low, (**A**) representative anti-NTSR1 WB performed after SDS-PAGE of SDS lysates from HEK293T cells overexpressing NTSR1 (HEK293T-NTSR1) or not (HEK293T-WT). HEK293T-NTSR1 were treated or not (untreated) 1 h with 100 nM Neurotensin alone or cotreated with the broad MMP inhibitor Marimastat. (**B**) Anti-NTSR1 WB quantification of NTSR1-low and NTSR1-high protein forms of HEK293T-NTSR1 cells presented in A. The NTSR1 signal is normalized to Na + /K + ATPase signal, before normalization to NTS alone treatment condition. + /- SEM, N = 3. (**C**) representative anti-NTSR1 WB performed after SDS-PAGE of SDS lysates from HEK293T cells overexpressing NTSR1 (HEK293T-NTSR1) or not (HEK293T-WT). HEK293T-NTSR1 were treated or not (untreated) 1 h with 100 nM Neurotensin alone or cotreated with the broad MMP inhibitor Batimastat. (**D**) Anti-NTSR1 WB quantification of NTSR1-low and NTSR1-high protein forms of HEK293T-NTSR1 cells presented in C. The NTSR1 signal is normalized to Na + /K + ATPase signal, before normalization to NTS alone treatment condition. + /- SEM, N = 3. *p < 0.05; **p < 0.01; ***p < 0.001. (**E**) representative anti-NTSR1 WB performed after SDS-PAGE of total GPCR fraction extracted from total membranes of HT29-KD (NTSR1 depleted) or HT29#3 (NTSR1-expressing) cells. HT29#3 were treated with vehicle or the broad MMP inhibitor Batimastat alone. Non-saturating higher exposure of the NTSR1-high signal is displayed. (**F**) Anti-NTSR1 WB quantification of NTSR1-low and NTSR1-high protein forms of HT29#3 cells presented in E. The NTSR1 signal is normalized to Na + /K + ATPase signal, before normalization to vehicle condition. + /- SEM, N = 3. (**G**) representative anti-NTSR1 WB performed after SDS-PAGE of total GPCR fraction extracted from total membranes of HT29#3 (NTSR1-expressing) cells. HT29#3 were treated with vehicle or the broad MMP inhibitor Marimastat alone. Non saturating higher exposure of the NTSR1-high signal is displayed. (**H**) Anti-NTSR1 WB quantification of NTSR1-low and NTSR1-high protein forms of HT29#3 cells presented in G. The NTSR1 signal is normalized to Na + /K + ATPase signal, before normalization to vehicle condition. + /- SEM, N = 3. *p < 0.05; **p < 0.01; ***p < 0.001. Uncropped WB blots images are included in Supplementary information.

To further characterize NTSR1 biology, we investigated the nature of the NTSR1-low form. As we have shown: (1) that NTS treatment induced NTSR1-low form to increase concomitantly with the decrease of the NTSR1-high form (Fig. 1B); (2) that unlike the NTSR1-high form, NTSR1-low form did not carry any glycosylation modification (Fig. 3A; suppl. Fig. 6A,B); (3) that NTSR1-low form apparent MW is lower than the NTSR1-LP form (Fig. 1); (4) that NTSR1-low form is neither a NTSR1 protein isoform nor an alternative splicing of NTSR1 mRNA (Fig. 1C,D), we therefore hypothesized that NTSR1-low form was produced by the cleavage of NTSR1-high’s N-terminal domain which carries the glycosylation modification (Fig. 1D). The persistence of the anti-NTSR1 antibody epitope and of the NTSR1 tags (GFP and FLAG), all located in the C-terminal portion of the protein, ascertain that the C-terminal domain of NTSR1 is not cleaved (Fig. 1E,F).

As NTSR1 is delivered to the cell surface and matrix associated metalloproteinases (MMP) are well known extracellular proteases, we reasoned that they could be involved in NTSR1 N-terminal domain cleavage^37–39^. We treated HEK293T-NTSR1 cells with NTS alone or co-treated with various concentrations of Marimastat or Batimastat, two broad spectrum MMP inhibitors (Fig. 4A,B,C,D, suppl. Fig. 7). NTS alone induces a decrease of the NTSR1-high form and an increase of the NTSR1-low form, whereas co-treatment with Marimastat or Batimastat restored NTSR1-high and NTSR1-low form levels to basal levels in a dose dependent manner (Fig. 4B,D, suppl. Fig. 7). These results demonstrate that the electrophoretic migration shift from the NTSR1-high to the NTSR1-low form is induced by MMPs activity supporting an extracellular N-terminal cleavage of NTSR1-high protein form to generate the NTSR1-low form.

To confirm the MMP dependent NTSR1 cleavage in tumoral cells endogenously expressing NTSR1, we evaluated the effect of MMP activity in HT29 cells (Fig. 4E,F,G,H). The cells were treated with Marimastat or Batimastat for 24 h before cell lysates were analyzed by WB. MMPs inhibition induced an increase of the NTSR1-high form and a decrease of the NTSR1-low form in a dose dependent manner (Fig. 4E,F,G,H). This demonstrates in tumor cells an exchange from the NTSR1-high form to the NTSR1-low form that depends on MMPs activity.

To confirm the physiopathological relevance of our findings, we analyzed NTSR1 expression in PDAC tumoral patient derived xenograft (PDX) models. NTSR1 expression was first evaluated and confirmed by qPCR and ^125^I-NTS binding approaches (suppl. Fig. 8). We were able to discriminate between NTSR1-expressing PDXs and non-expressing samples, which were used as negative controls (suppl. Fig. 8). Only the NTSR1-low form was identified by WB indicating that the NTSR1-low form was the most abundant form in PDAC PDX samples. As we have shown that the NTSR1 low form is the product of NTSR1-high form, this result supports the in vivo processing of NTSR1 by MMPs (suppl. Fig. 8)*.*

Altogether, these results support that NTSR1 is cleaved by MMPs to form the NTSR1-low form in non-tumoral and in tumoral context, in vitro and in vivo, and that this cleavage is potentiated by NTS.

### NTSR1 protein internalization and NTSR1-low form degradation are promoted by NTS

**Figure 5:**
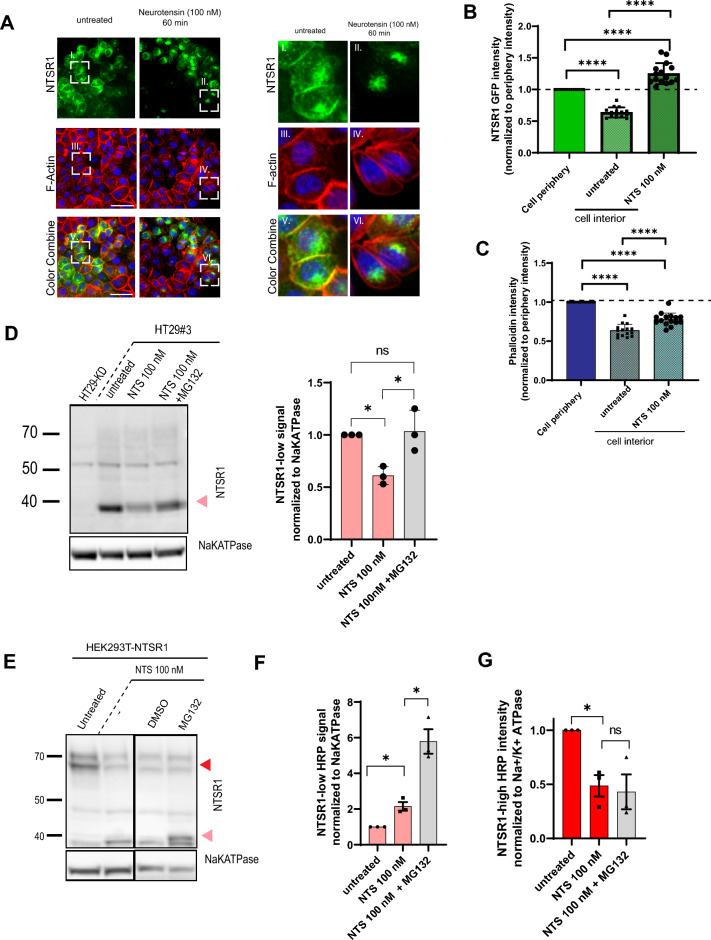
Neurotensin promotes NTSR1’s internalization and the degradation of NTSR1 low form by the proteasome. (**D**, **E**) Arrowheads: Red: NTSR1-high, pink: NTSR1-low, (**A**) Representative confocal images of PANC1 cells overexpressing the C-terminal GFP-tagged NTSR1. Cells were treated or not (untreated) with 100 nM of Neurotensin for 1 h before being fixed and stained with Phalloidin (F-actin) and with Hoechst. (**B**) Quantification of NTSR1 internalization. Plasma membrane (cell periphery) and cytoplasmic (cell interior) NTSR1-GFP were measured in PANC1-NTSR1GFP treated or not (untreated) with NTS 100 nM for 1 h (NTS 100 nM), and normalized to NTSR1-GFP cell periphery intensity. (**C**) Quantification of F-actin localization as a control. Plasma membrane (cell periphery) and cytoplasmic (cell interior) F-actin were measured in PANC1-NTSR1GFP treated or not (untreated) with NTS 100 nM for 1 h (NTS 100 nM) and normalized to cell periphery intensity. (**D**) Left, Representative anti-NTSR1 WB performed after SDS-PAGE of total GPCR fraction extracted from total membranes of HT29-KD (NTSR1 depleted), HT29#3 (HT29 clone). HT29#3 were treated or not (untreated) with 100 nM of Neurotensin for 1 h alone (NTS 100 nM) or co-treated 3 h with the proteasome inhibitor MG132 (2 µg/mL). HT29-KD are used as negative control. Right, Anti-NTSR1 WB quantification of NTSR1-low protein forms presented on the left. The NTSR1 signal is normalized to Na + /K + ATPase signal, before normalization to untreated condition. + /- SEM, N = 3. (**E**) Representative anti-NTSR1 WB performed after SDS-PAGE of SDS lysates from HEK293T cells overexpressing NTSR1 (HEK293T-NTSR1) treated or not (untreated) with DMSO or 1 h with 100 nM Neurotensin alone or cotreated with the proteasome inhibitor MG132 (2 µg/mL). (**F**) Anti-NTSR1 WB quantification of NTSR1-low protein form presented in E. The NTSR1 signal is normalized to Na + /K + ATPase signal, before normalization to untreated condition. + /- SEM, N = 3. (**G**) Anti-NTSR1 WB quantification of NTSR1-high protein form presented in E. The NTSR1 signal is normalized to Na + /K + ATPase signal, before normalization to untreated condition. + /- SEM, N = 3, *p < 0.05; ****p > 0.0001. Uncropped WB blots images are included in Supplementary information.

We have shown that in response to NTS, the NTSR1-low form is degraded (Fig. 2C). To identify the responsible degradation pathway, we first investigated NTSR1 localization in response to NTS treatment and found that the receptor was rapidly internalized, as its plasma membrane localization was lost (Fig. 5A,B,C). However, the lysosomal degradation pathway was not involved in NTSR1 degradation following internalization as lysosomal acidification inhibition by Chloroquine did not rescue the protein degradation induced by NTS (suppl. Fig. 9). Therefore, we suspected the proteasomal degradation pathway to be involved. To test our hypothesis, we treated HT29#3 cells with NTS alone or in combination with MG132, a proteasomal inhibitor (Fig. 5D). MG132 treatment was able to rescue the decrease of the NTSR1-low form that was induced by NTS. Proteasomal degradation pathway activity is therefore responsible for the NTS-induced NTSR1-low degradation.

To confirm that only the NTSR1-low form was degraded, we investigated NTSR1-high form abundance following co-treatment with NTS and MG132 in HEK293T-NTSR1 cells which express high levels of NTSR1-high (Fig. 5E,G). Whereas NTS alone induced the decrease of NTSR1-high and an increase of NTSR1-low, co-treatment with MG132 did not rescue the decrease of the NTSR1-high, showing that NTSR1-high form is not degraded by the proteasome. In line with our findings in HT29 and PANC1 cells, MG132 addition promoted an even greater increase of NTSR1-low form in comparison to NTS treatment alone (Fig. 5F).

Altogether, these results demonstrate that only the N-terminally cleaved NTSR1-low form is degraded by the proteasome upon internalization.

## Discussion

**Figure 6:**
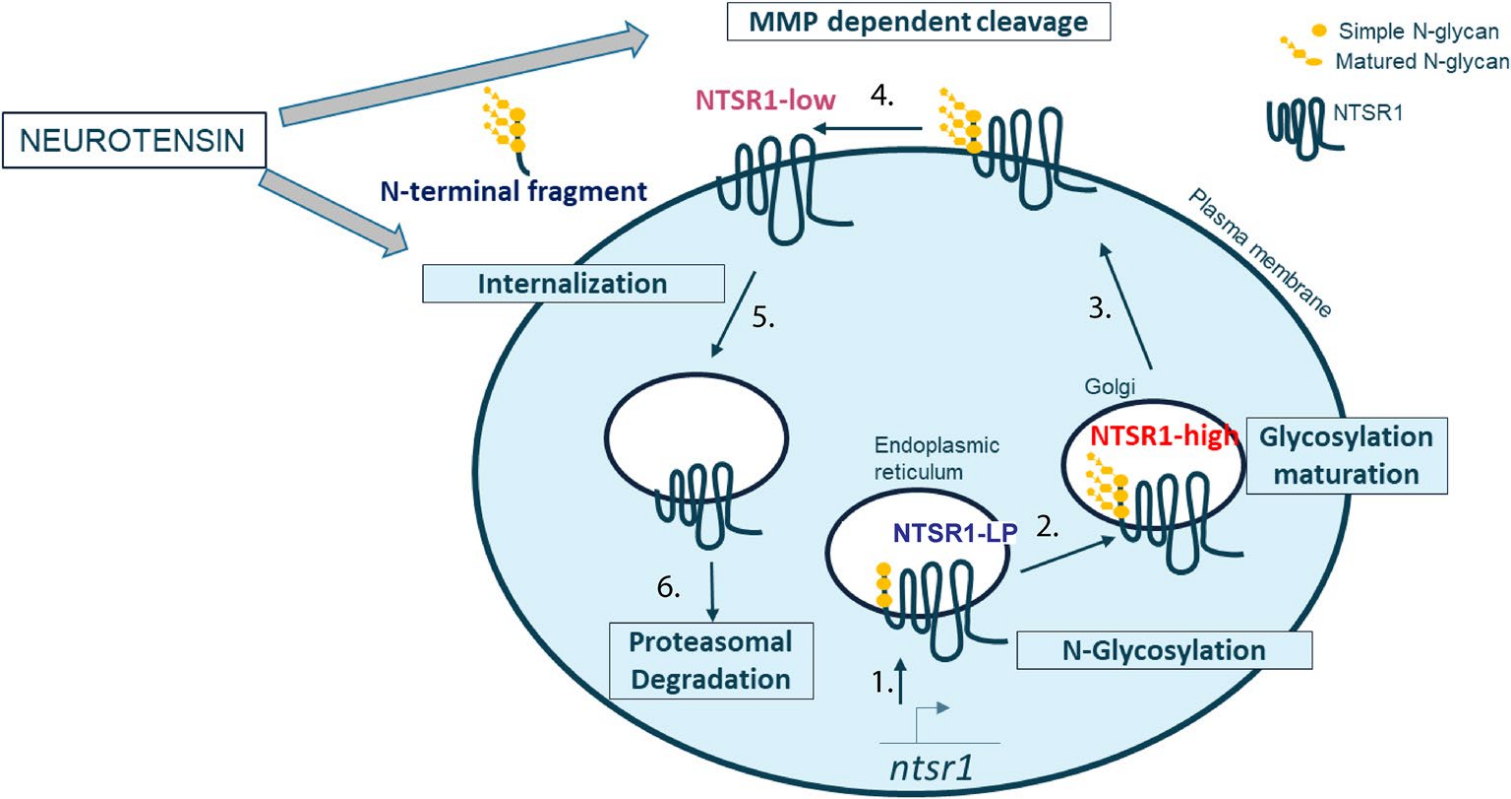
NTSR1 protein biology’s graphical summary. Illustration of NTSR1 biology: 1-NTSR1 is expressed as the NTSR1-LP and co-translationally glycosylated in the ER (Fig. 3); 2-NTSR1 is transported and processed in the Golgi to form the NTSR1-high form Fig. 3; 3-NTSR1 is delivered to the plasma membrane (Figs. 3,5); 4-NTSR-high is cleaved by MMP to form the NTSR1-low protein form (Fig. 4); 5-NTS promotes NTSR1 internalization (Fig. 5); 6-NTSR-low form is degraded by the proteasome (Fig. 5)

Our work characterizes NTSR1 protein biology, showing that the protein exists in three forms through post translational modifications: Glycosylation of the NTSR1-LP form produces the NTSR1-high form while MMP dependent cleavage produces the NTSR1-low form, which lacks the N-terminal glycosylated extracellular domain (Fig. 6).

Glycosylation is a common PTM of GPCRs^25,38^ known to contribute to the protein’s stability and localization at the plasma membrane^26,40^. We report for the first time glycosylation of NTSR1 protein and identify the resulting protein form, NTSR1-high, which displays a mobility shift in PAGE of around 20 kDa in comparison to NTSR1-LP, the unglycosylated protein. In the case of NTSR1 protein, it seems glycosylation supports the stability of NTSR1 as we have shown that NTSR1-low, the N-terminally cleaved and unglycosylated protein form, is degraded by the proteasome whereas NTSR1-high was not affected.

By contrast, the PTM responsible for NTSR1-low form expression, MMP dependent cleavage, is rarely observed and has been described for only seven other class A GPCRs^37,41–46^. More frequent investigation of this type of PTM may reveal its greater contribution to the biology of class A GPCRs. Here we show that the MMP cleavage produces two fragments of NTSR1, a N-terminal fragment that is assumed to be shed to the extracellular space and the C-terminal fragment, i.e. NTSR1-low, that remains in cell membranes. Indeed, this was similarly shown for the GPR37 GCPR by Mattila et al. who were able to retrieve the shed N-terminal domain of that MMP-cleaved GPCR and demonstrated its glycosylated nature by submitting it to PNGase-F treatment^38,47^. As NTSR1’s glycosylated residues are located in the extracellular N-terminal domain of the protein, N-terminal shedding also removes NTSR1’s glycosylation motifs making MMP cleavage a de-glycosylating mechanism for NTSR1 and resulting in NTSR1-high and NTSR1-low colocalizaton at the plasma membrane.

Of note, we have shown that NTSR1-low formation is promoted by NTS, the NTSR1 agonist ligand, and also found NTSR1-low to be the most abundant form of NTSR1 in our cancer cells and PDX models. NTSR1-low form may therefore be the mediator of the NTS/NTSR1 protumoral signaling activity. In line with this hypothesis, MMP dependent cleavage of PAR1 and Sphingosine-1-P receptor GPCRs was found to induce GPCR activation in tumoral context^41,48^. Although an increase of systemic NTS ligand levels was measured in patients with gastric cancer in comparison to healthy patients^23^ and NTS was found to promote the expression and activation of MMPs^23,24,49^, further investigation is needed to understand the regulation of the MMP-dependent NTSR1 cleavage.

Until now, most studies have investigated NTSR1 biology and contribution to tumorigenesis using NTSR1-overexpressing non-tumoral cells. However, we show that in contrast to cancer cells, which express more NTSR1-low, HEK293T cells overexpressing NTSR1 expressed more abundantly the NTSR1-high form. We find that increased expression of NTSR1 protein causes impairment of protein trafficking leading to its accumulation at the Golgi, where the protein may remain uncleaved as only plasma membrane-localized NTSR1-high proteins are accessible to MMP, causing NTSR1-high form intracellular accumulation. Expression of MMPs and of the NTS ligand have been found to be increased in tumor cells in comparison to the non-tumoral HEK293T-NTSR1 cells^23^, which would lead to lower NTSR1 cleavage and further contribute to NTSR1-high form accumulation in NTSR1-overexpressing non-tumoral cells. Therefore, until the function and contribution of the different NTSR1 forms to NTS/NTSR1 signaling are determined, NTSR1-overexpressing cell models should be used with caution when investigating NTSR1 protein biology.

In response to NTS ligand, NTSR1 is internalized and NTSR1-low but not NTSR1-high is degraded by the proteasome. NTSR1-high is therefore either recycled back to the plasma membrane as reported by Law et al.^50–53^ or the Golgi-accumulated NTSR1-high is the source of the plasma membrane recovery, a process that would be independent of receptor recycling or protein translation^50,51,53^. To discriminate between these two scenarios, tools allowing the monitoring of the endogenous NTSR1 protein localization should be developed. Because of the absence of its N-terminal extremity and of its glycosylation modification, NTSR1-low may be identified as a defective NTSR1 protein form by the proteasome leading to its degradation.

New NTSR1-targeting strategies such as NTSR1-vectorized antitumoral therapies rely on NTSR1 protein availability and accessibility at the cancer cell surface^21^. Based on our findings, promotion of these features can be achieved by limiting cleavage of NTSR1 by MMPs. Additionally, to target the protumoral NTS/NTSR1 signaling pathway more efficiently, it is crucial to understand the specific roles of NTSR1-low and NTSR1-high forms. It is necessary to determine whether both forms of the protein require NTS binding for their signaling activity or whether the NTSR1-low form is constitutively active, in which case Meclinertant/SR48692, the NTSR1-antagonizing small molecule, would not be efficient in tumors that we found to express more abundantly the NTSR1-low form (Akter et al., 2015; Körner et al., 2015; Moody et al., 2014, NCT00290953). Finally, it would be interesting to evaluate the biomarker potential of the shed NTSR1 N-terminal fragment by detecting its presence in the systemic circulation or in the peritumoral environment^38,47,54^.

## Methods

### Live animal experiments/PDX

All procedures using live animals and methods were carried out in accordance with relevant guidelines and regulations and all methods are reported in accordance with ARRIVE guidelines. The experiments were approved by the Ethical Committee (CEEA) no 91 (Oncomet) and no 31 (Ipsen Innovation) and were authorized by the French Ministry of Research.

Pancreatic PDX tumors were acquired from Oncodesign and stored frozen at − 80 °C.

### Source of cell lines

HEK293T and HEK293T-NTSR1 cell lines were purchased from trenzyme (Trenzyme, #1564). HEK293T-NTSR1 stable cell line was generated with the transduction into the HEK293T cells of an optimized-mutated sequence of NTSR1 that translated into the normal amino acid sequence of NTSR1. PANC1 (ATCC, CRL-1469) and HT29 (ATCC, HTB38) were purchased from ATCC.

### Cell culture conditions

HEK293T, HEK293T-NTSR1 and PANC1 were cultured in DMEM (4.5 g/L). HT29 cells were cultured in McCoy’s medium. All media were supplemented with FBS (10%), Pyruvate (1 mM) and Glutamax (2 mM). The cells were cultured in a humified controlled atmosphere (37 °C, 5% CO_2_).

### Reagents

Neurotensin (4006469, Bachem), PNGase F (Thermofisher, A39245), Tunicamycin (Sigma, SML1287), Marimastat (Sigma, M2699), Batimastat (Tocris 2961), MG132 (Sigma, M7449), Monensin (00-4505-51, eBioscience) and Chloroquine (C6628-sigma) were diluted in cell culture medium to treat the cell culture.

### Lentiviruses and cell transduction

To generate the HT29-shNTSR1, the pGIPZ lentiviral particles expressing NTSR1 targeting shRNAs were obtained from dharmacon and stored at − 80 °C. There reference and sequences are indicated: shRNA#068 (V3LHS_646268; 5′TTATTGAGAGATACACGGG3′), shRNA#54 (V3LHS_643354; 5′ATATGAAGGACATGAAGGT3′), shRNA#48 (V3LHS_643348; 5′TGACCTGTATGACGACCTT3′).

To obtain the HEK293T-GFP, HEK293T-NTSR1GFP, HEK293T-NTSR1FLAG respectively, the pLV lentiviral particles expressing GFP alone (pLV[Exp]-Puro-CMV > EGFP), NTSR1-GFP (pLV[Exp]-Puro-CMV > EGFP/ hNTSR1[NM_002531.3] (VB200409-1087krk) and NTSR1-flag (: pLV[Exp]-PuroCMV > hNTSR1[NM_002531.3]/FLAG) were obtained from Vectorbuilder and stored at − 80 °C.

For cell transduction, viral particles were added to the cell suspension (2 × 10^5^ cells/mL) in 500 µL complete medium supplemented with protamine (8 µL) at a MOI = 2 for HT29 and PANC1 and at a MOI = 1 for HEK293T. Cells were left to adhere and grow for 3 days before trypsinization and culture as needed.

### siRNA transfection

Custom designed Ambion siRNA targeting the optimized-NTSR1 sequence were ordered from thermofisher with the following sequence:

- 5′-TACGTGAGTTCCACCATAAAT
- 5′-ACTGTCTGTGGAAAGATATTT
- 5′-AGCGTGCTGAACACCATTATA

HEK293T-NTSR1 cells were transfected with the Lipofectamine RNAimax (thermofisher) following the manufacturer recommendations.

### CrisPR cas9 gene editing

To obtain the polyclonal HT29-Crispr-NTSR1, HT29 cells were transduced with the lentiviral plasmids pRLP.U6.sg1NTSR1 containing the NTSR1 targeting guide RNA sequences SG1 (3′CAACGCTTCGGGCAACGCGT5′) and the pRLP.EF1.CAS9WT expressing the cas9 protein. To validate the efficient indels induced by the cas9, the targeted *ntsr1* nucleotide sequence was amplified by PCR, sequenced and analyzed by the TIDE software. The polyclonal population was subcloned by limit dilution and the NTSR1-knock down (KD) HT29-KD clone was isolated. The control clone HT29#3 clone was isolated from the polyclonal HT29 population processed with the control RNA guide tomato.

### Cell treatment

Cells were grown in complete medium for 3 days; the medium was removed, and cells were put in presence of the appropriate treatment and processed for WB or IF analysis. *NTS treatment* NTS (100 nM) diluted in complete medium was added to the cells and incubated at 37 °C for 1 h and cells were processed with appropriate assay. *Tunicamycin treatment* Cells were incubated with Tunicamycin (2 µg/mL) diluted in complete medium for 24 h. *MG132 treatment* cells were incubated for 2 h in complete medium containing MG132 (2 µg/mL) alone, before an hour incubation in complete medium supplemented with both NTS (100 nM) and MG132 (2 µg/mL).* Batimastat/Marimastat treatment* HEK293T-NTSR1 cells were incubated for 2 h with Batimastat or Marimastat alone before addition of NTS and MG132 (2 µg/mL). HT29 cells were incubated for 24 h with Batimastat or Marimastat alone and cells were processed for WB analysis.

### Cell's total membranes preparation

The following steps are performed on ice. Briefly, adherent cells were rinsed twice with ice-cold PBS (1X), detached by scraping in PBS, pelleted by centrifugation (500 g, 4 °C), rinsed with Tris–HCl (50 mM, pH 7,4) and pelleted again by centrifugation (500 g, 4 °C). The cell pellet was resuspended in Tris–HCl (50 mM, pH 7,4) and lysed by sonication (amplitude 28%, 4 × 10 s, on ice). Total membrane was pelleted by ultra-centrifugation (46 000 g, 10 min, 4 °C), and the supernatant, which contained the cytosolic fraction, was discarded.

### NTS binding assay Saturation

^125^I-NTS Binding was assessed in microconic tubes in a 400 µL volume: 30 µg of cell membranes were mixed in Tris–HCl (50 mM, pH 7,4) supplemented with BSA (0.2%) with a concentration range of ^125^I-NTS (0 to 4 nM) and left to incubate for 60 min. To assess the non-specific^125^I-NTS binding the same binding mix was supplemented with cold NTS (10 µM). The binding reactions were terminated by filtration onto GF/C filter plate (PerkinElmer, 6005174) with a harvester filtration system (Filtermate Harvester Packard, PerkinElmer), followed by washes with Tris–HCL (50 mM, pH 7.4) and allowed to dry. MicroScint-0 (20 µL, PerkinElmer, 6013611) was added to each well and ^125^I radioactivity was measured on TopCount (Packard, PerkinElmer, NXT). The Bmax is calculated as follow M = count in cpm / 10-9/2200 Ci/mmol/0.6/2.2/400 µL/decay factor then calculated Bmax value in fmol/mg = concentration in M*0.0004*10^15^/mg cell membrane proteins.

### SDS Cell Lysis

To prepare the SDS cell lysis samples, adherent cells were rinsed twice with ice-cold PBS-CM (1X, thermofisher, 14040133), scraped in SDS lysis buffer (2% SDS, 50 mM, Tris–HCl, pH 8,3) and the cell lysate was harvested and sonicated (amplitude 28%, 4 × 10 s) to decrease sample viscosity.

### GPCR extraction

GPCR extracts were prepared either from total cell fraction or from only the membrane fraction:

#### From total cell

The following steps are performed on ice. Briefly, adherent cells were rinsed twice with ice-cold PBS-M (1X, thermofisher, 14040133), detached by scraping in PBS-CM, pelleted by centrifugation (500 g, 4 °C). The GPCR fraction was extracted by resuspension in the GPCR extraction reagent, following manufacturer recommendations.

#### From total membranes

The GPCR fraction was extracted from the total membrane fraction using GPCR extraction reagent (thermofisher, A43436) following the manufacturer protocol. Briefly, total membrane pellet was resuspended in GPCR extraction reagent supplemented with phosphatase et protases inhibitors (thermofisher 78,442), incubated under shaking for 1 h and centrifuged (13 000 g, 45 min, 4 °C). The supernatant that contained the GPCR fraction was kept and the pellet was discarded.

### SDS-PAGE- Nitrocellulose transfer-immunoblotting

Equal amount of proteins were incubated in sample buffer (1X, thermofisher, B0007) supplemented with reducing agent (1X, thermofisher). The samples were subjected to SDS–polyacrylamide gel electrophoresis (PAGE) using precast acrylamide gels (Thermofisher, NP0335BOX), and proteins were transferred to nitrocellulose membranes using nitrocellulose transfer stack (thermofisher, IB301031). Membranes were incubated for 60 min in Blocking solution (Tris-buffered saline (TBS) containing 0.1% Tween-20, 5% milk) and further incubated overnight with the appropriate primary antibody diluted in Blocking solution at 4 °C. The membranes were then washed three times with TBS(1X)–Tween (0.1%) and incubated for 60 min with secondary antibody conjugated to horseradish peroxidase (HRP). Bound antibodies were detected with enhanced chemiluminescence (thermofisher SuperSignal West Dura, 34076). The following primary antibodies were used at the indicated dilutions: rabbit anti-NTSR1 (1/5000e thermofisher, PA3-214), anti- Na/KATPase-HRP (1/5000e abcam, ab185065), anti-GFP (1/1000e abcam, ab290), anti-flag (1/1000e clone M1, sigma, F3040).

### Blot acquisition and quantification

WB HRP signal was acquired with the pixie 4 (Syngene) imaging system and quantified with the GeneTool analysis software. For each condition testing, the bands intensities were normalized to the corresponding loading control before normalization to control condition of the test.

### Immunofluorescence

Adherent cells were rinsed twice with PBS-CM (1X, thermofisher, 14040133), fixed with PFA (4%, PBS-CM 1X) 10 min at room temperature and permeabilized with saponin (0,1%) containing Permeabilization buffer (1X, eBioscience, 00-8333) 10 min at room temperature. Samples were incubated with primary antibodies diluted in 10% FBS, PBS (1X) overnight at 4 °C, then washed with PBS and incubated with secondary antibodies diluted in 10% FBS, PBS (1X), 1 h at room temperature. The following primary antibodies, secondary antibodies and staining molecules were used at the corresponding dilution: anti-NTSR1 (1/100e sigma, SAB43000718), anti-GM130(1/200e, Abcam, ab52649), anti-rabbit Cy3, Phalloidin Alexa fluor 594 (1:200e, thermofisher, A12381), Hoechst (thermofisher, 33258).

The images were acquired with the ImageXpress Micro Confocal Imaging system (Molecular Devices) and quantified with imageJ. For NTSR1 internalization quantification, NTSR1-GFP was measured at the cell periphery (20-pixel wide delimitation around the phalloidin staining) and cell’s interior NTSR1-GFP intensity (. Each NTSR1-GFP cell interior intensity was normalized to its respective cell periphery NTSR1-GFP. Phalloidin intensity measurement was used as control.

### FACS + method of quantification

HEK293T-GFP or HEK293T-NTSR1-GFP were allowed to grow for 3 days, the medium was removed and the cells rinsed 2 times with ice-cold PBS, before being detached with Versene (15,040,066, Gibco Thermofisher). Cells were stained with the anti-NTSR1 BN-6 antibody (1 µg/1 × 10^6^ cells; abcam ab269711) labelled to R-PE with the conjugation kit Mix-n-stain (cliniscience, 92299) following the manufacturer recommendations. HEK293T-GFP were used as NTSR1-expression negative control and to control the non-specific immunostaining. Over 50000 cells per condition were analyzed by the cytometer Fortessa X20-4 laser BD.

## Supplementary Information

Supplementary Information.

Supplementary Information 2.

## Data Availability

The *ntsr1*-optimized sequence generated and used in the current study is available in the Genbank repository, OQ366525.
