# Supplementary material for: NTSR1 glycosylation and MMP dependent cleavage generate three distinct forms of the protein
Source: Sci Rep. 2023 Mar 22;13:4663. doi: 10.1038/s41598-023-31790-7 (PMC10033925; doi:10.1038/s41598-023-31790-7)
Supplement: Supplementary file 1 — Supplementary Information. [file 41598_2023_31790_MOESM1_ESM.pdf]

Supplementary figure 1: NTSR1 SDS/PAGE WB optimal conditions

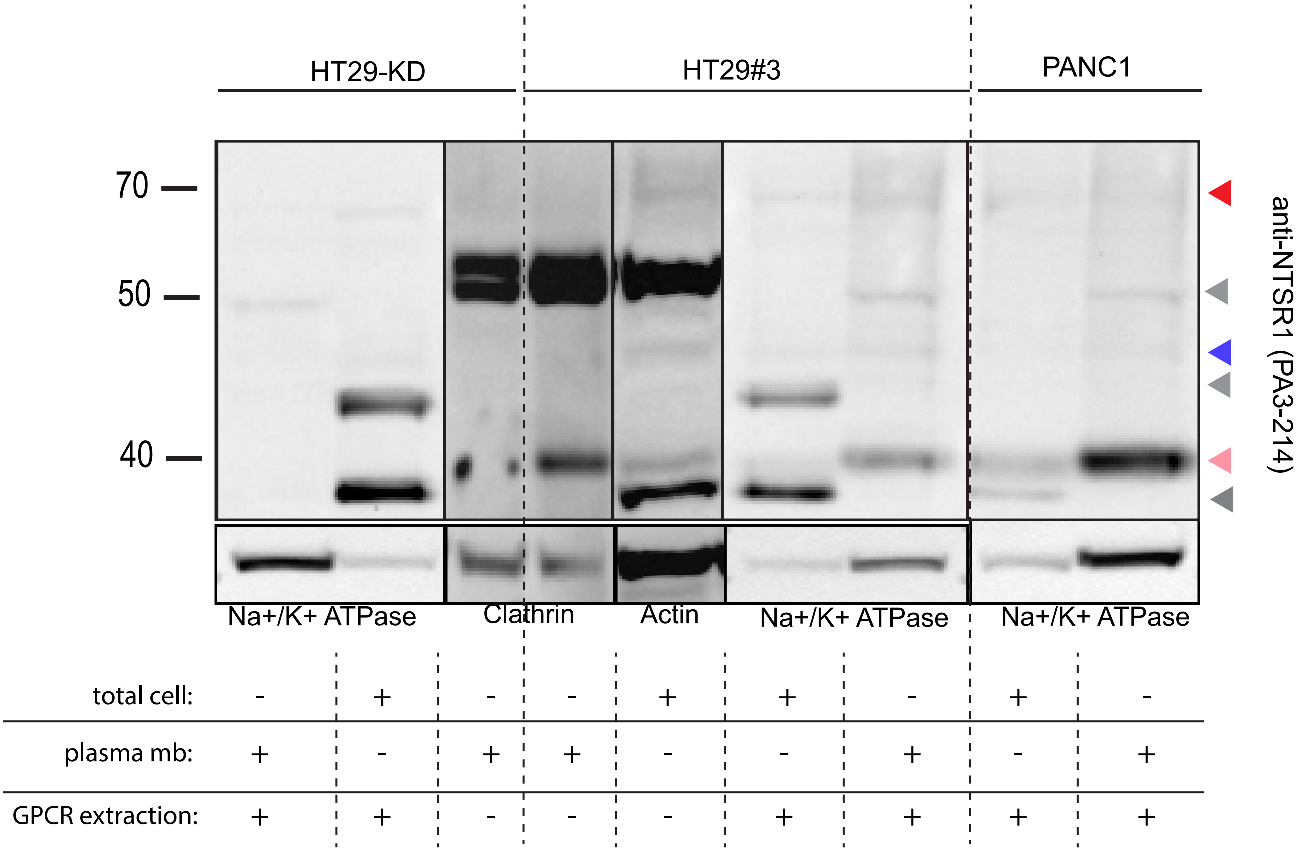

Anti-NTSR1 WB of HT29-KD, HT29#3 and PANC1 cells that have undergone total cell extraction or plasma membrane extraction alone or GPCR extraction from total cell extract or GPCR extraction from plasma membrane, before SDS-PAGE. Arrowheads: Red: NTS-R1-high, pink: NTSR1-low, blue: NTSR1-LP, grey: nonspecific band

Uncropped WB images are included at the end of the supplementary information.

Supplementary figure 2 : NTSR1 protein depletion by siRNA, shRNA and CRISPR-cas9

A

NTSR1 sgRNAs targets
NTSR1 shRNAs targets
Oprimised NTSR1 sequence siRNAs targets

```
hNTSR1      0  ATGCGCCTCAACAGCTCCGCGCCGGGAACCCCGGGCACGCCGGCCGCCGACCCCTTCCAG  60
optimised   0  atgaggctgaatagtagcgcccctggcactccgggcacgcctgcggctgatcccttccag  60
               *** * ** ** **   *** ** ** ** *********** ** ** ** *********

hNTSR1         CGGGCGCAGGCCGGACTGGAGGAGGCGCTGCTGGCCCCGGGCTTCGGCAACGCTTCGGGC  120
optimised      agagcccaagctggactggaggaagcgctgttggctcctgggttcggcaacgcttctgga  120
                * ** ** ** *********** ****** **** ** ** ************** **

hNTSR1         AACGCGTCGGAGCGCGTCCTGGCGGCACCCAGCAGCGAGCTGGACGTGAACACCGACATC  180
optimised      aacgcgtctgaaagggtcttggccgcaccaagctccgagttggatgtcaacaccgacatc  180
               ******** **  * *** **** ***** ***  **** **** ** ************

                                         ¦

hNTSR1         AGCCTGAGTGTGGAGCGCTACCTGGCCATCTGCCACCCCTTCAAGGCCAAGACCCTCATG  540
optimised      tcactgtctgtggaaagatatttggccatctgccacccgttcaaagccaaaactcttatg  540
                  ***  ******  * **  **************** ***** ***** ** ** ***

                                         ¦

hNTSR1         GGCCTGGTGTGCACCCCCACCATCCACACTGCCACCGTCAAGGTCGTCATACAGGTCAAC  720
optimised      ggtctggtttgtacgcccaccatccatacggccacggtgaaagtcgtgatacaagtaaat  720
               ** ***** ** ** *********** ** ***** ** ** ***** ***** ** **

hNTSR1         ACCTTCATGTCCTTCATATTCCCCATGGTGGTCATCTCGGTCCTGAACACCATCATCGCC  780
optimised      acgttcatgagcttcatcttccccatggttgtgataagcgtgctgaacaccattatagcc  780
               ** ******  ****** *********** ** **    ** *********** ** ***

                                         ¦

hNTSR1         GTGCGGCGCCTCATGTTCTGCTACATCTCGGATGAGCAGTGGACTCCGTTCCTCTATGAC  1020
optimised      gttagaaggctcatgttctgctacatcagcgacgaacaatggaccccattcctgtacgac  1020
               **  *  * *****************   ** ** ** ***** ** ***** ** ***

hNTSR1         TTCTACCACTACTTCTACATGGTGACCAACGCACTCTTCTACGTCAGCTCCACCATCAAC  1080
optimised      ttctatcactactttacatggtgaccaatgccctgttctacgtgagttccaccataaat  1080
               ***** ******** ************** ** ** ******** ** ******** **

                                         ¦

hNTSR1         AGCGTGTCCAGCAACCACACCCTCTCCAGCAATGCCACCCGCGAGACGCTGTACTAG     1257
optimised      agtgtgagcagcaatcatacacttagctcaaatgcgacaagggagactttgtactaa     1257
               ** ***  ****** ** ** **    *    ***** **  * *****  *******
```

B

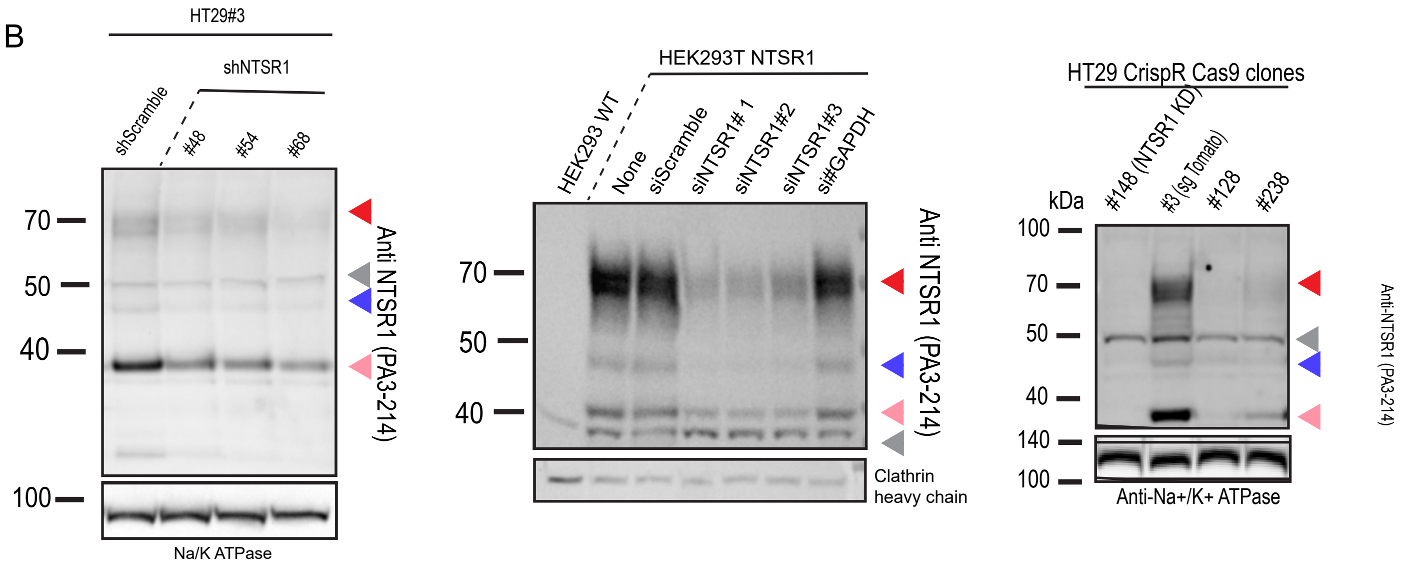

A, Alignment of human NTSR1 and optimized NTSR1 cDNA sequence. NTSR1 sgRNA sequence, shRNA sequences and siRNA are highlighted respectively in blue, brown and red.
B, Arrowheads: Red: NTSR1-high, pink: NTSR1-low, blue: NTSR1-LP, grey: nonspecific band
B, Left, anti-NTSR1 WB of HT29#3 cells transduced with control scrambled shNRA (shScramble) or NTSR1 shRNAs (#48, #54, #68). Middle, anti-NTSR1 WB of HEK293 WT or HEK293T-NTSR1 transfected with control scramble siRNA (shScramble) or GAPDH siRNA (si#GAPDH) or NTSR1 siRNAs (siNTSR1#1,2,3). Right, Uncropped WB images are included at the end of the supplementary information.

Supplementary figure 3 : NTSR1 protein construct

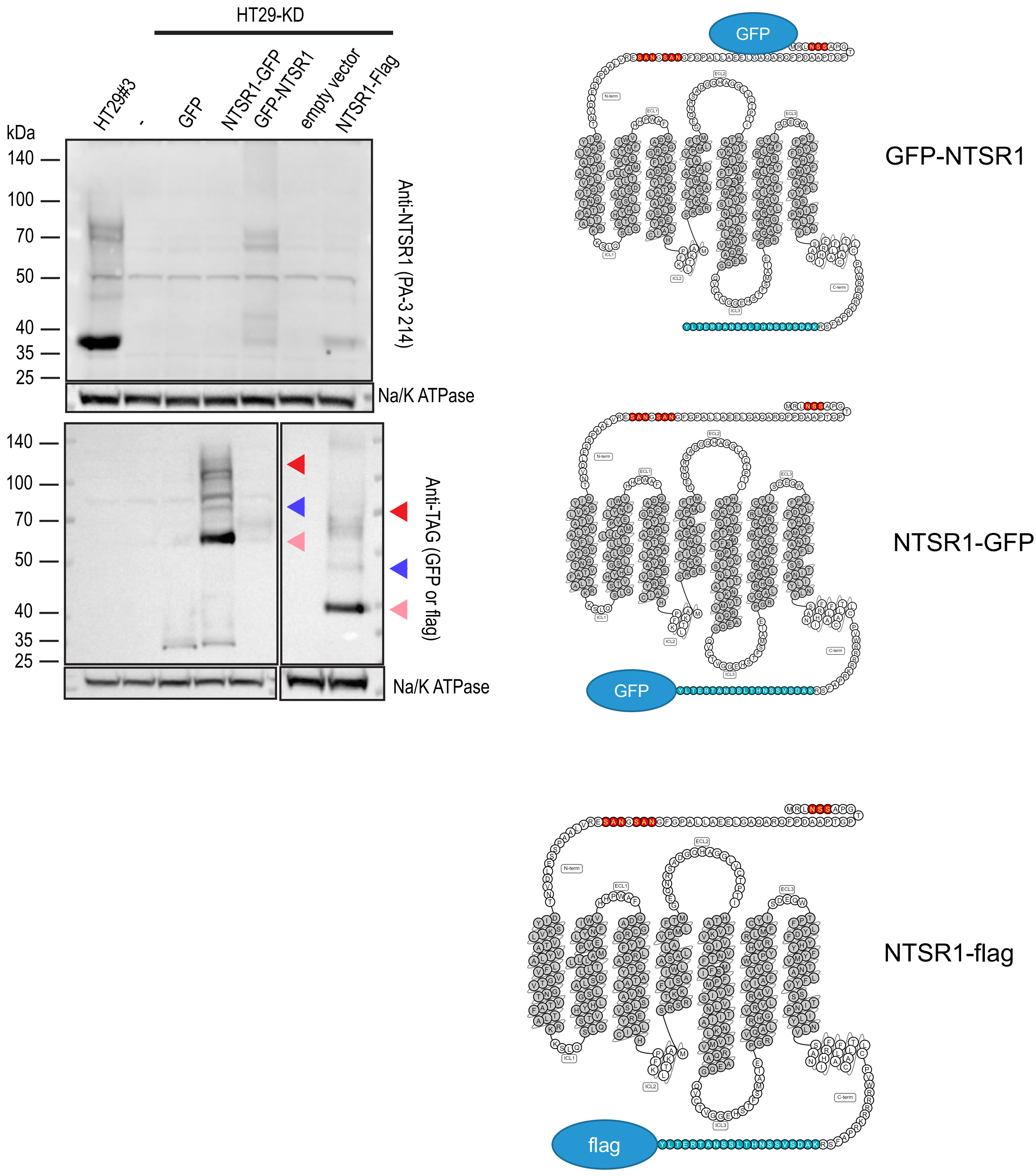

Anti-NTSR1 or Anti-TAG WB of HEK293T cells overexpressing NTSR1 tag constructs. Snake diagrams of the NTSR1 constructs are presented on the right.
Arrowheads: Red: NTSR1-high, pink: NTSR1-low, blue: NTSR1-LP,
Uncropped WB images are included at the end of the supplementary information.

Supplementary figure 4 : Neurotensin dose response effect on NTSR1 forms expression

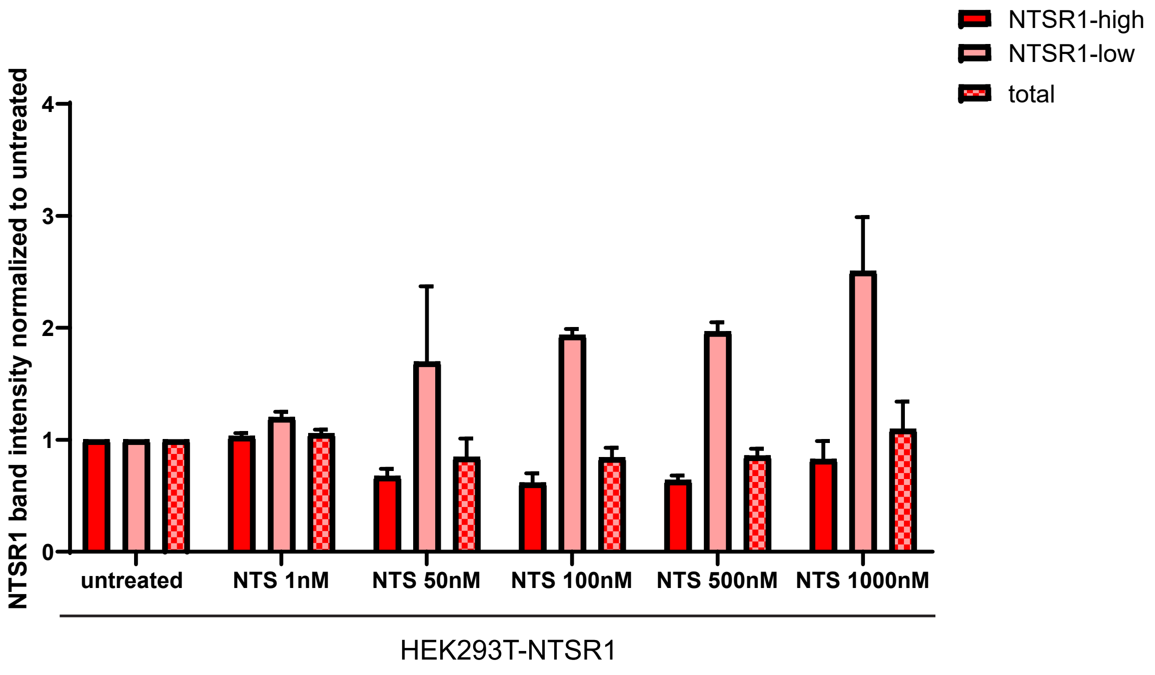

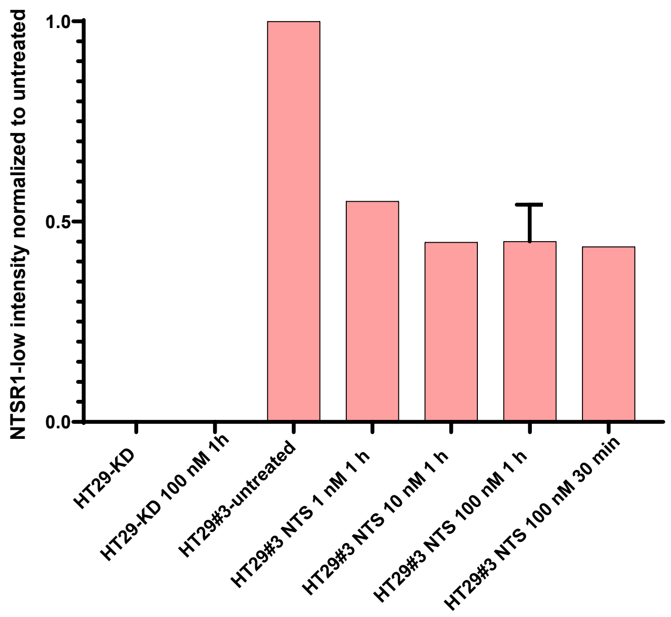

Upper panel: Quantification of anti-NTSR1 WB of HEK293T-NTSR1 treated with increasing concentration of NTS for 1 hour. Lower Panel: Quantification of anti-NTSR1 WB of HT29 treated with increasing concentration of NTS for 1 hour.

Supplementary figure 5 : NTSR1-high is not destabilized by denaturing agents

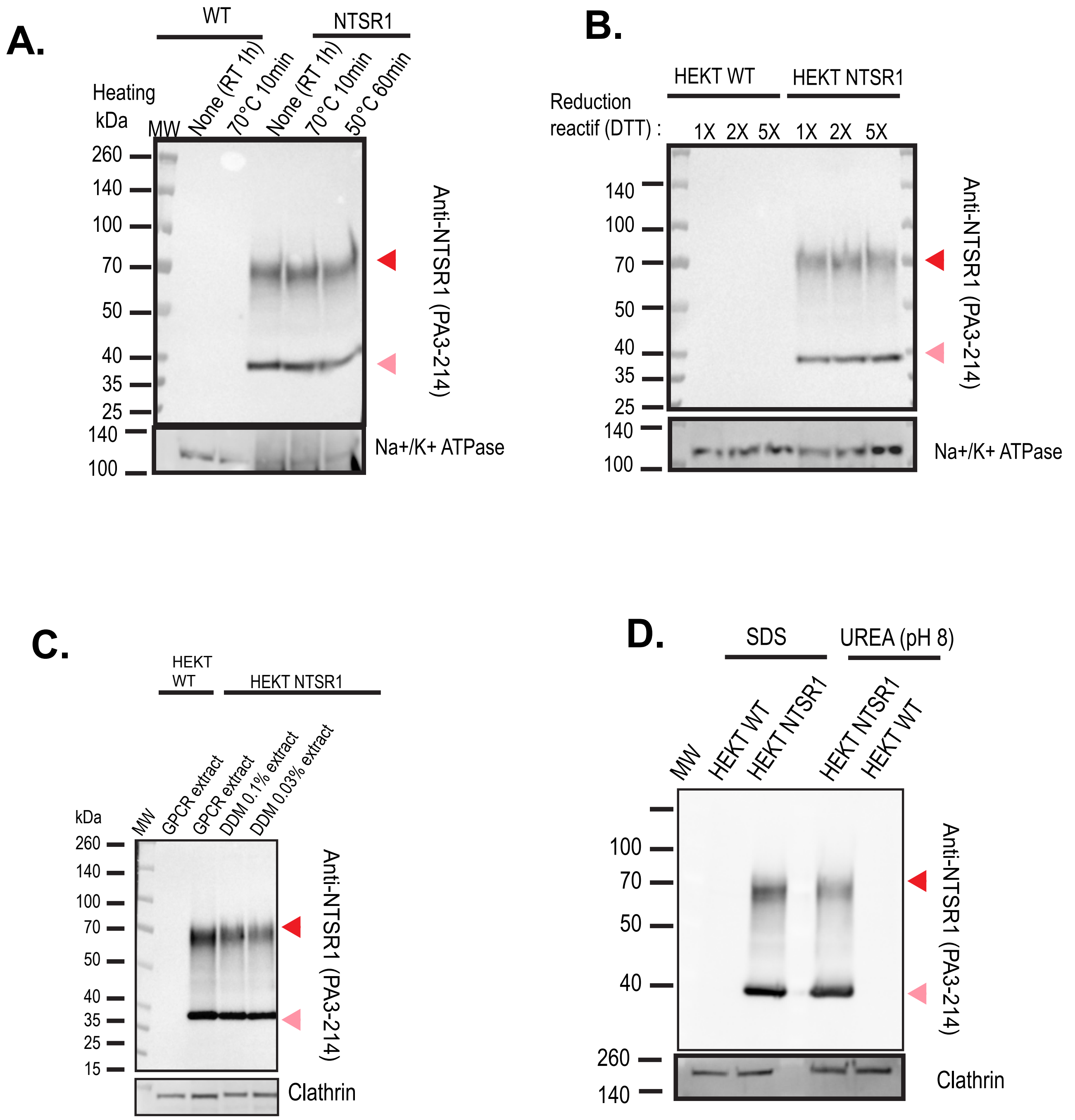

Anti-NTSR1 WB of HEK293T WT or HEK293T-NTSR1 GPCR extracts from plasma membrane extraction A, that have undergone heating (50°C or 70°C) for 10 min or 1 hour or no heating (RT, 1 hour) B, that were treated with 1X, 2X or 4X of the reduction agent, C, that were treated with non-ionic denaturing agent DDM (0.1% or 0.03%) D, that were treated with ionic denaturing agent SDS and Urea 6 M.

Uncropped WB images are included at the end of the supplementary information.

Supplementary figure 6 : NTSR1 is deglycosylated by PNGase F and BADG

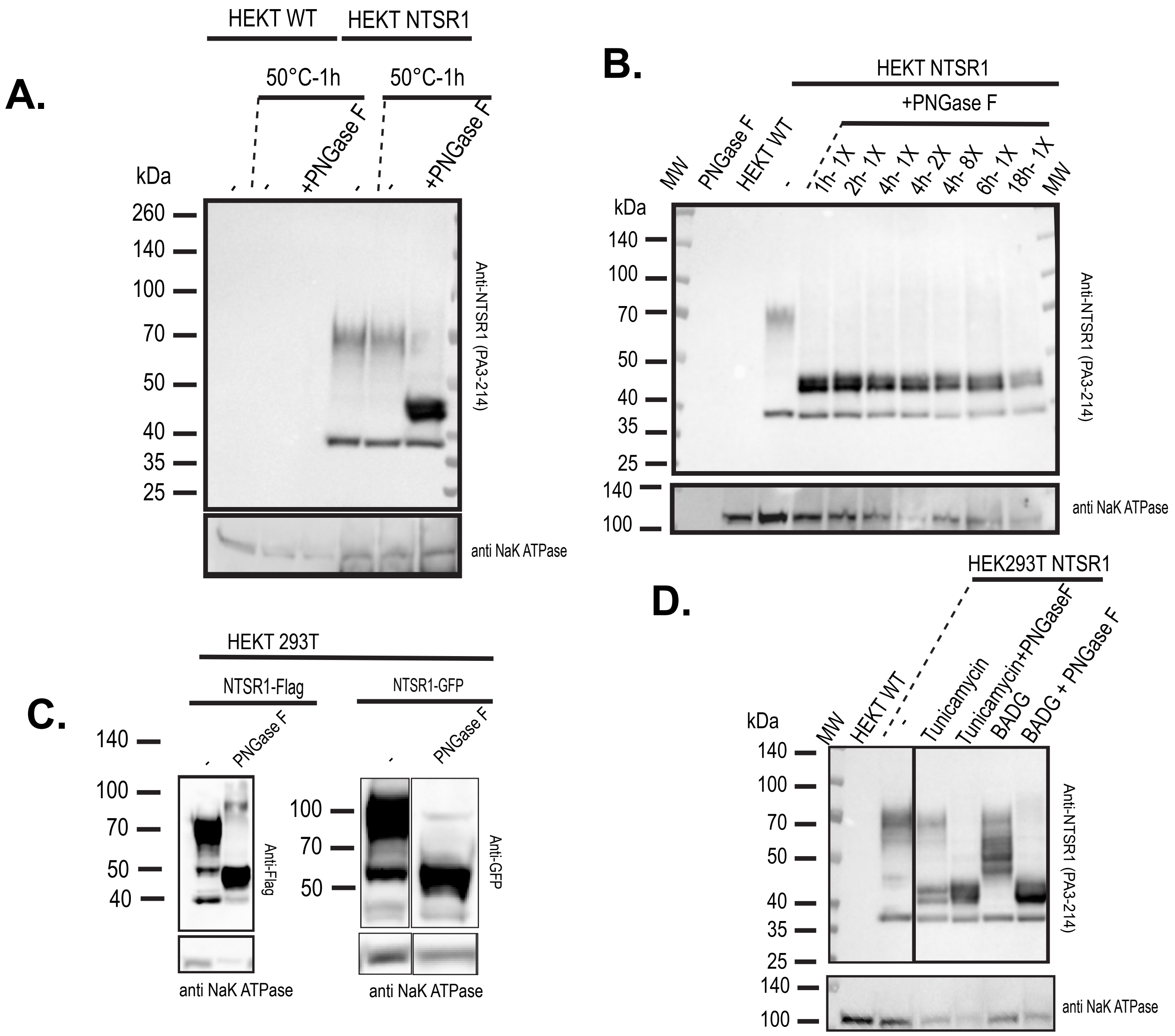

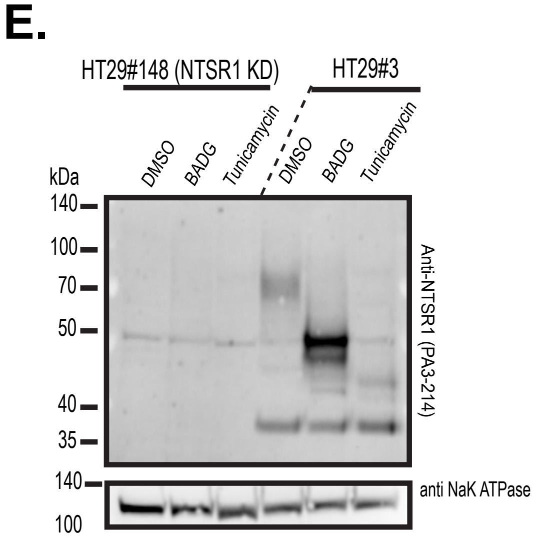

Anti-NTSR1 WB of HEK293T WT or HEK293T-NTSR1 GPCR extracts from plasma membrane extraction A, heated for 1 hour alone or with PNGase F, B, treated with different concentration of PNGase Fand incubation. C, Anti-FLAG or anti-GFP WB performed on HEK293T WT or overexpressing NTSR1-FLAG or NTSR1-GFP, treated or not with the deglycosylase PNGase F. D, Anti-NTSR1 WB of HEK293T WT or HEK293T-NTSR1 GPCR extracts treated with Tunicamycin alone or with PNGase, or treated with BADG alone or with

Uncropped WB images are included at the end of the supplementary information.

Supplementary figure 7 : Uncropped WB from main figure 4

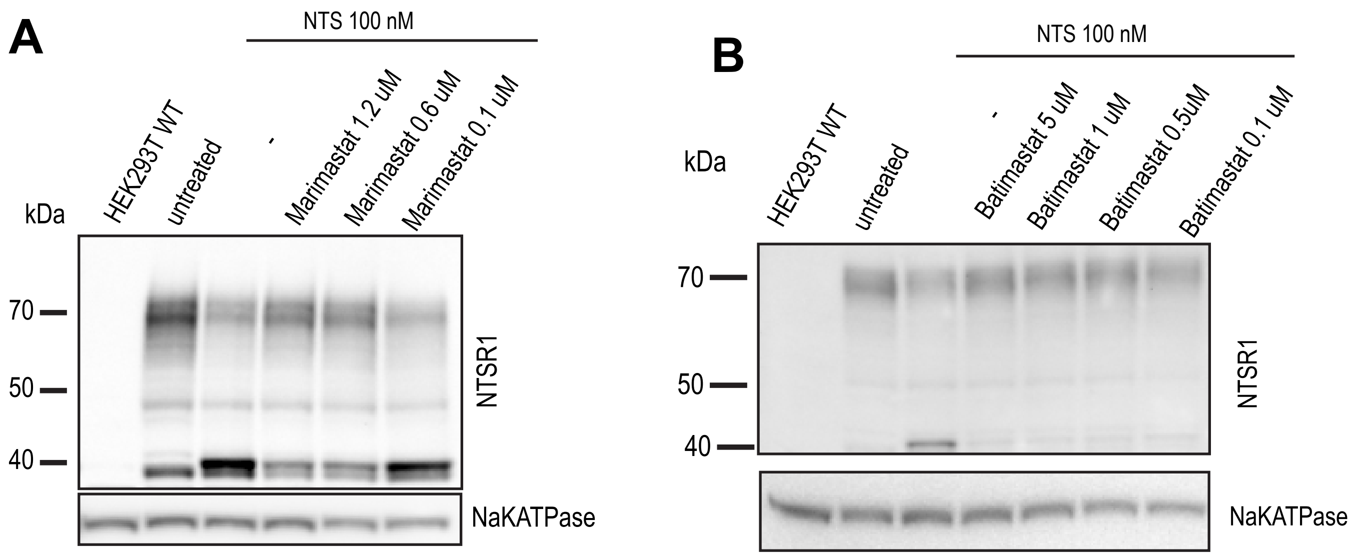

A, representative anti-NTSR1 WB performed after SDS-PAGE of SDS lysates from HEK293T cells overexpressing NTSR1 (HEK293T-NTSR1) or not (WT). HEK293T-NTSR1 were treated or not (untreated) 1 h with 100 nM Neurotensin alone or cotreated with decreasing concentration of the broad MMP inhibitor Marimastat.
B, representative anti-NTSR1 WB performed after SDS-PAGE of SDS lysates from HEK293T cells overexpressing NTSR1 (HEK293T-NTSR1) or not (WT). HEK293T-NTSR1 were treated or not (untreated) 1 h with 100 nM Neurotensin alone or cotreated with decreasing concentration of the broad MMP inhibitor Batimastat

Supplementary figure 8 : NTSR1 expression in CRC PDX

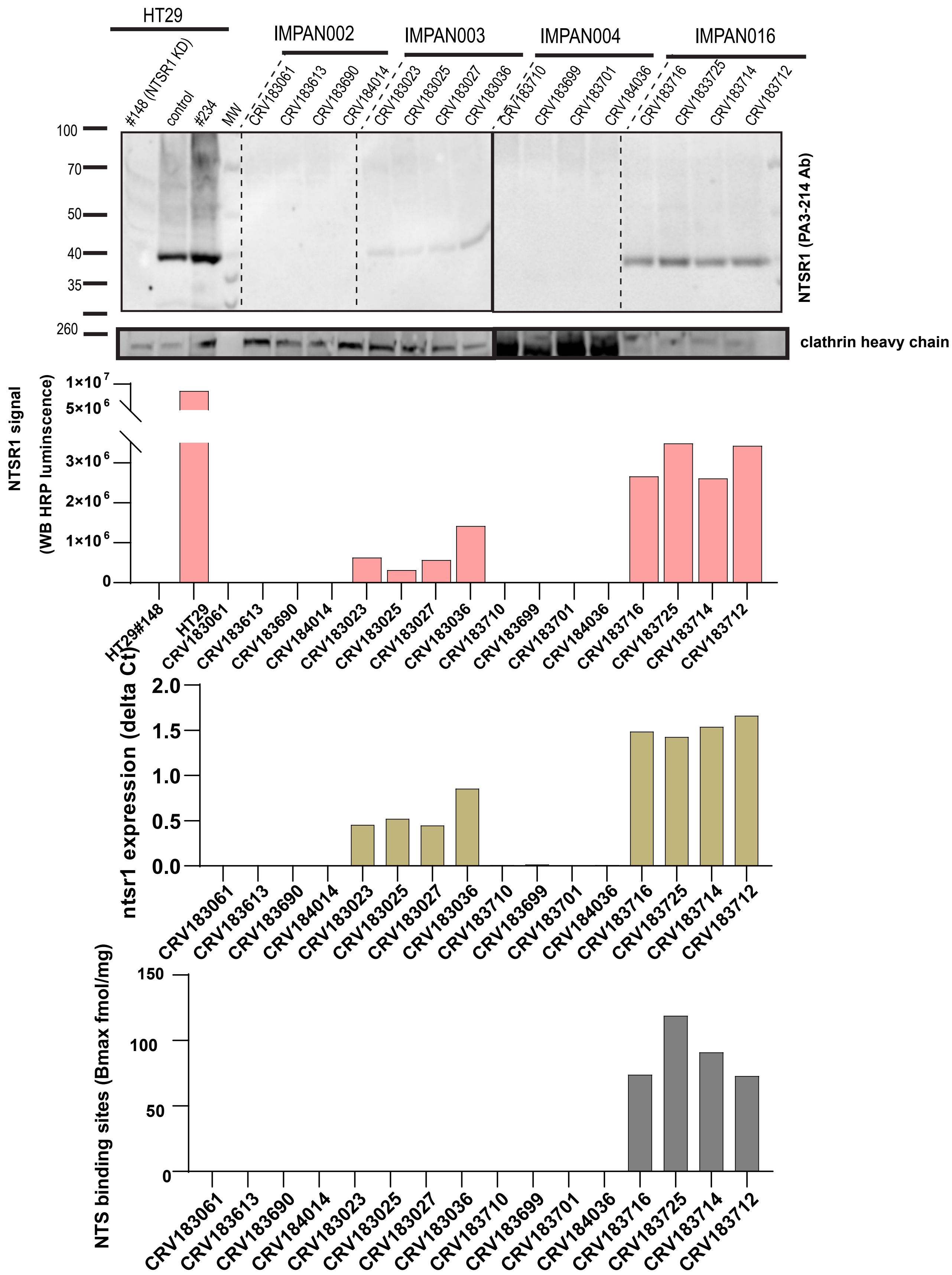

upper panel anti-NTSR1 WB on GPCR extract of PDAC PDX. HT29 GPCR extract were used as positive controls. Pink graph, quantification of NTSR1-low WB signal. Green graph, ntsr1 expression evaluated by qPCR on RNA extract of the PDAC PDX. Grey Graph, NTS binding performed on membrane extracted from the PDAC PDX.

Uncropped WB images are included at the end of the supplementary information.

Supplementary Figure 9 : Lysosomal degradation is not involved in NTSR1-low form degradation

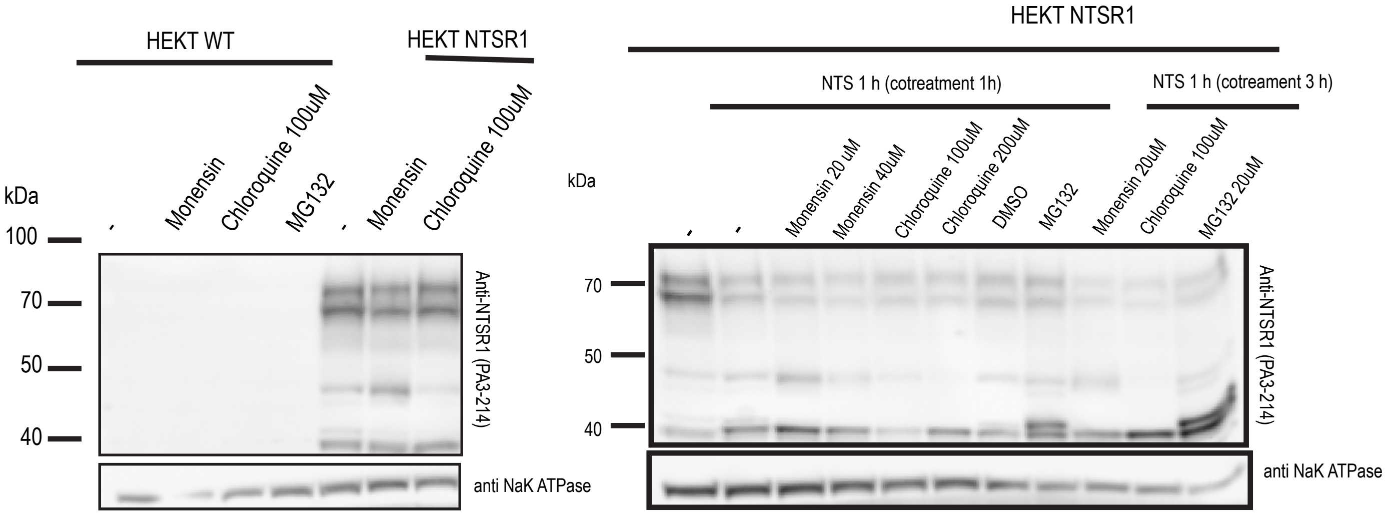

Anti-NTSR1 WB performed on HEK293T WT or HEK293T-NTSR1 treated with the lysosomal acidification inhibitors Monensin and Chloroquine or the proteasomal inhibitor MG132 alone or wo-treated with NTS 100 nM for 1 h.

Uncropped WB images are included at the end of the supplementary information.
