# Supplementary material for: NTSR1 glycosylation and MMP dependent cleavage generate three distinct forms of the protein
Source: Sci Rep. 2023 Mar 22;13:4663. doi: 10.1038/s41598-023-31790-7 (PMC10033925; doi:10.1038/s41598-023-31790-7)

Figure 1 : NTSR1 is expressed as 3 protein forms of different molecular weight

A

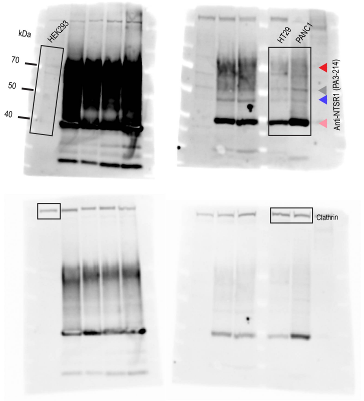

B

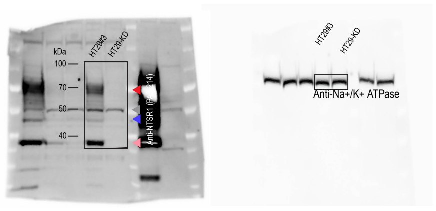

C

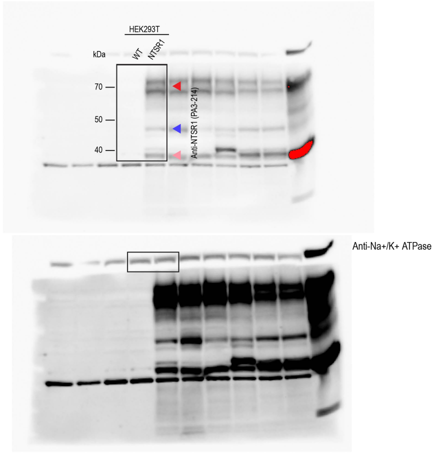

E,left

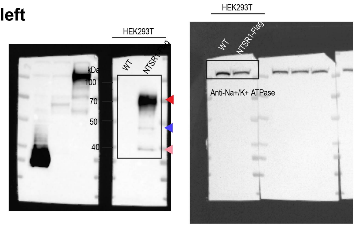

E, right

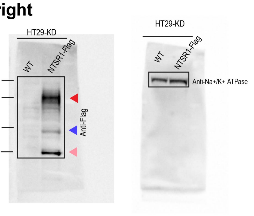

F, left

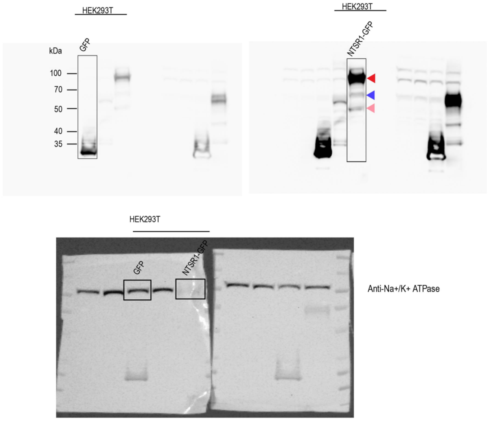

F, right

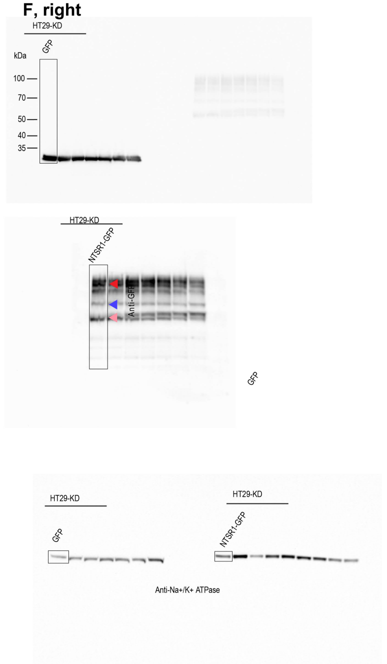

Figure 2 : Neurotensin regulates the relative abundance and stability of NTSR1 protein forms

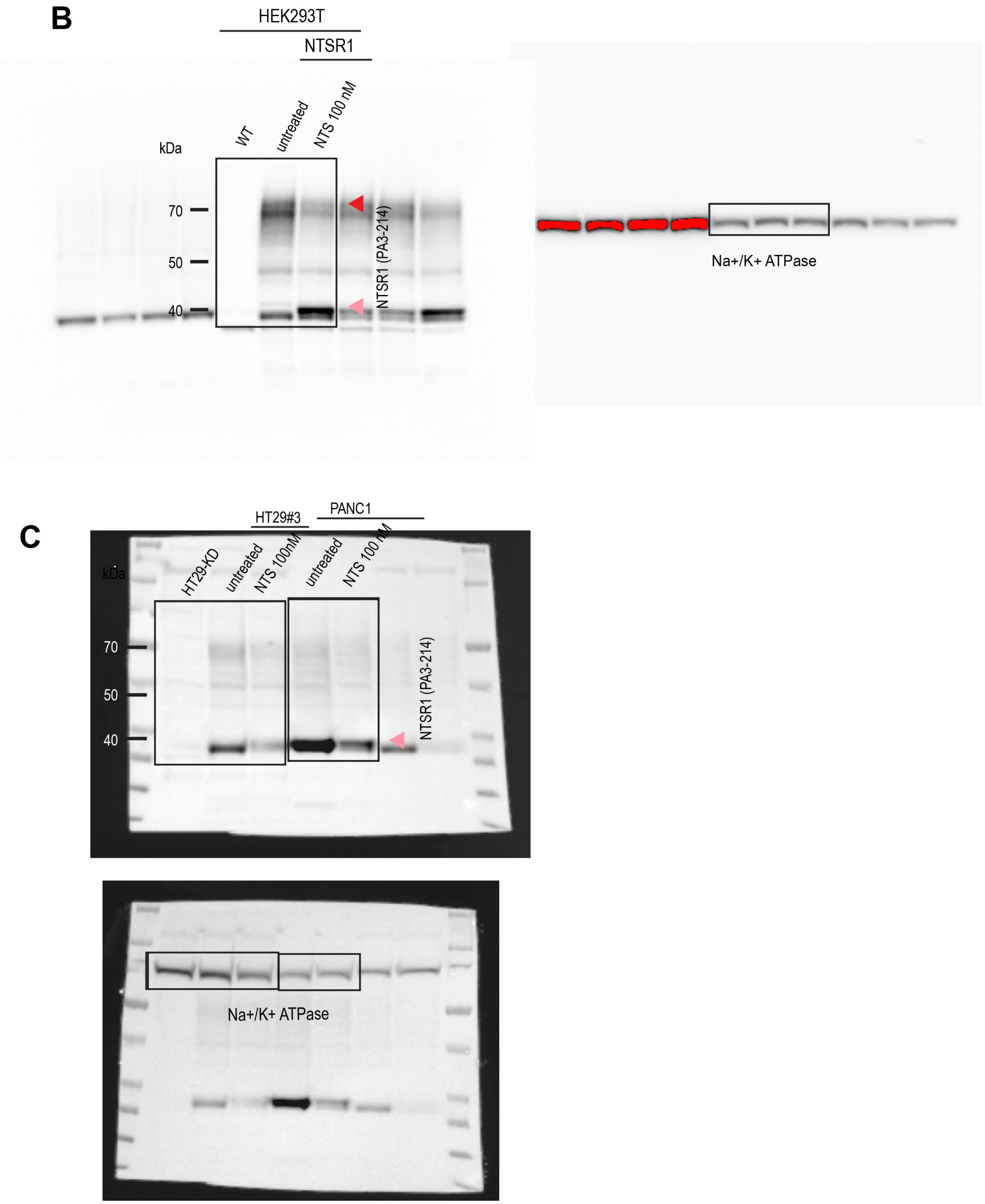

Figure 3 :Glycosylation of NTSR1 through the secretory pathway generates NTSR1-high form

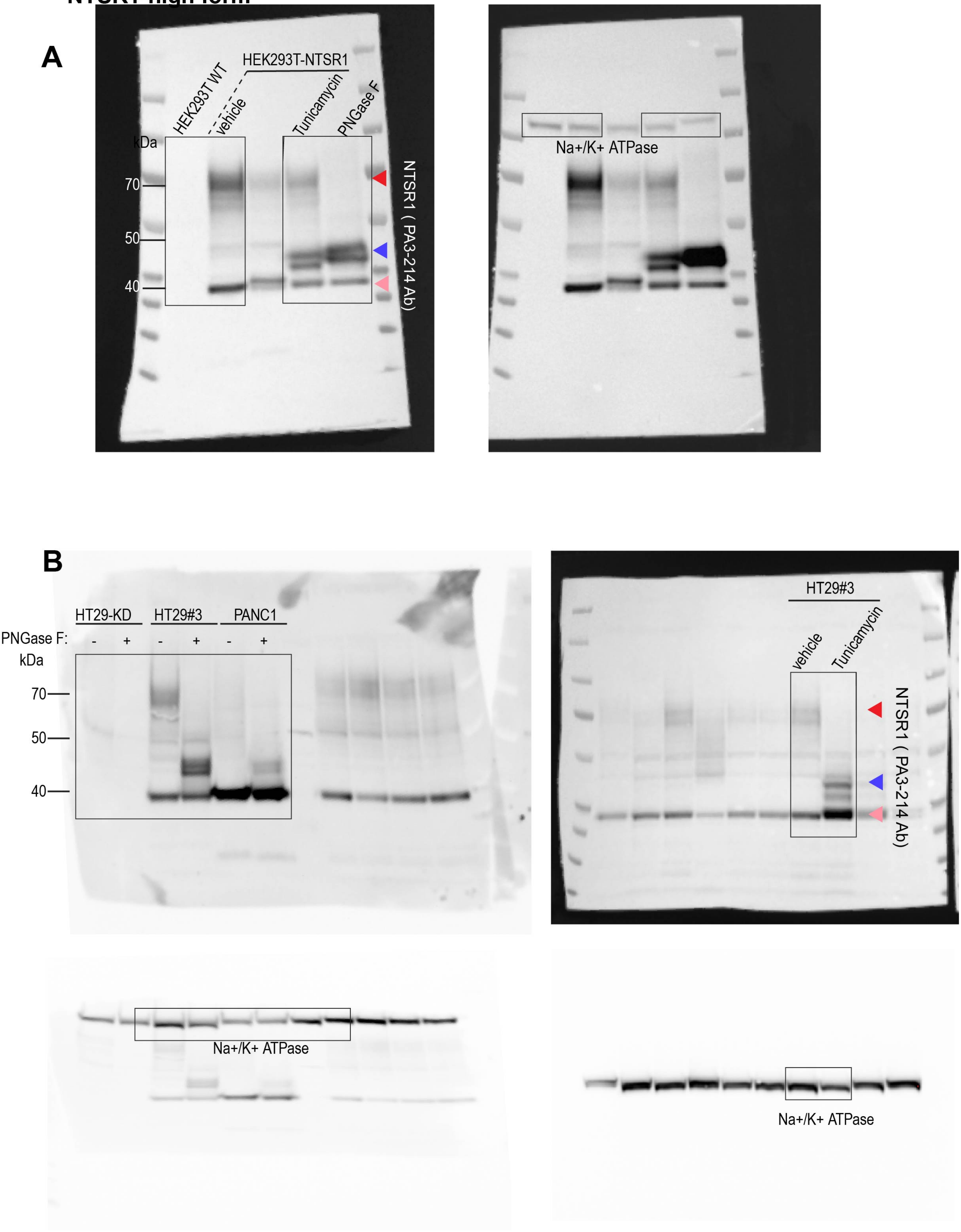

Figure 4: NTSR1-high form is N-terminally cleaved by matrix metalloproteinase to form the NTSR1-low protein form

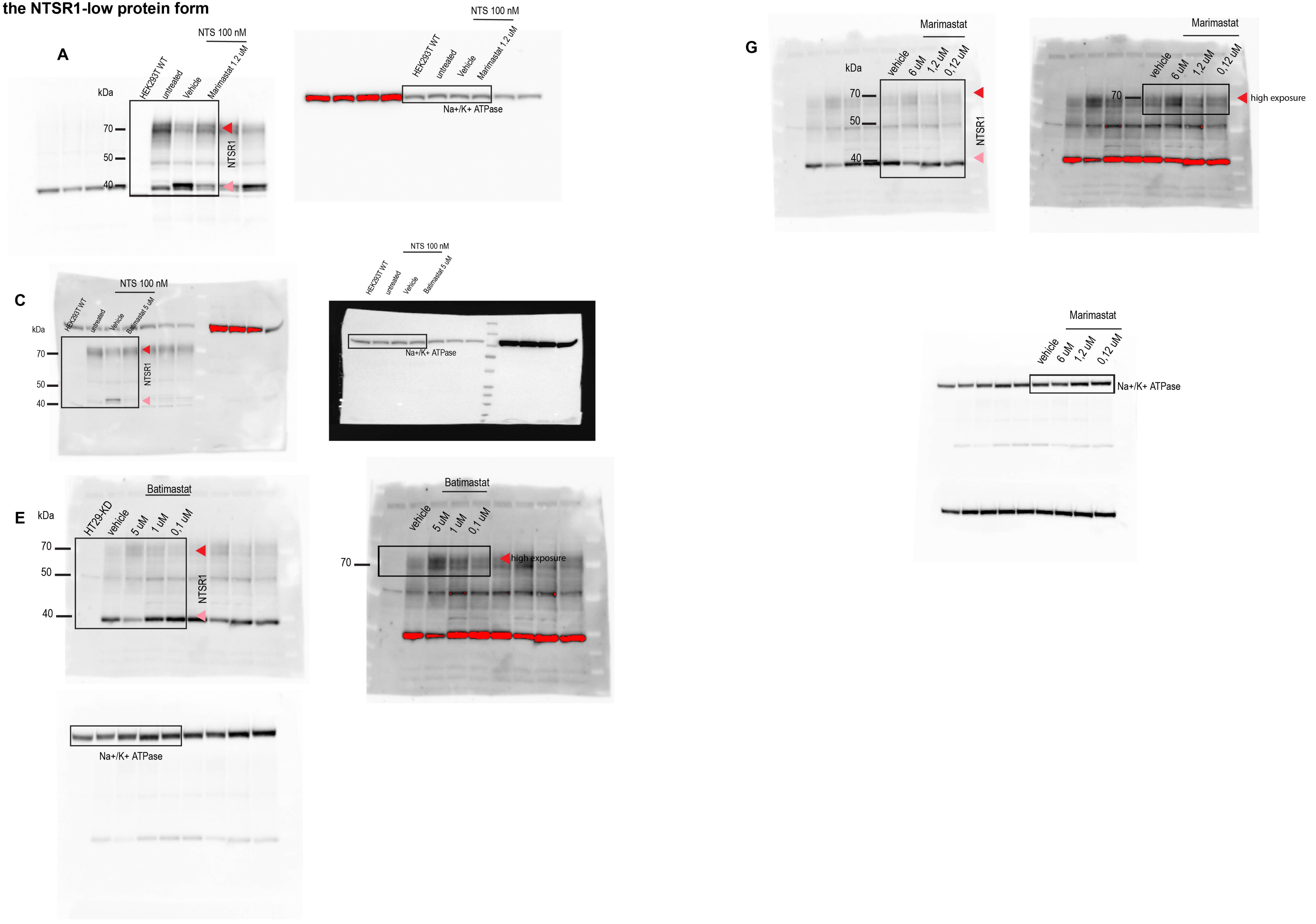

Figure 5: Neurotensin promotes NTSR1's internalization and the degradation of NTSR1 low form by the proteasome

D

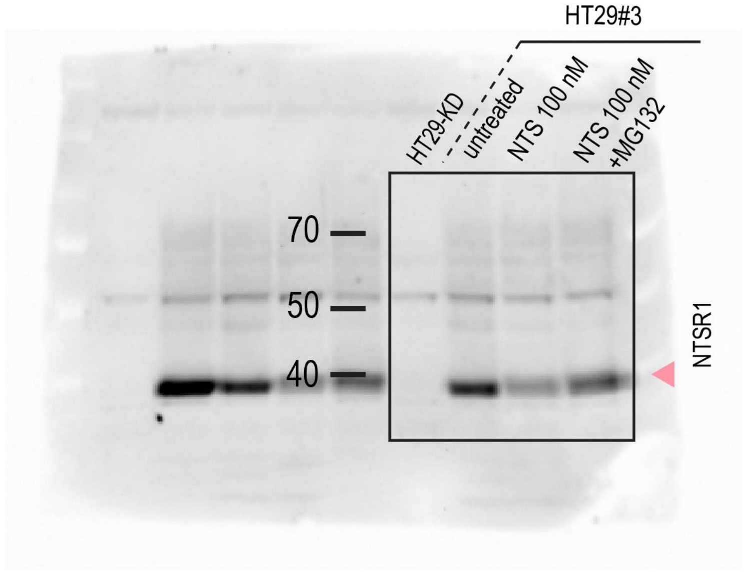

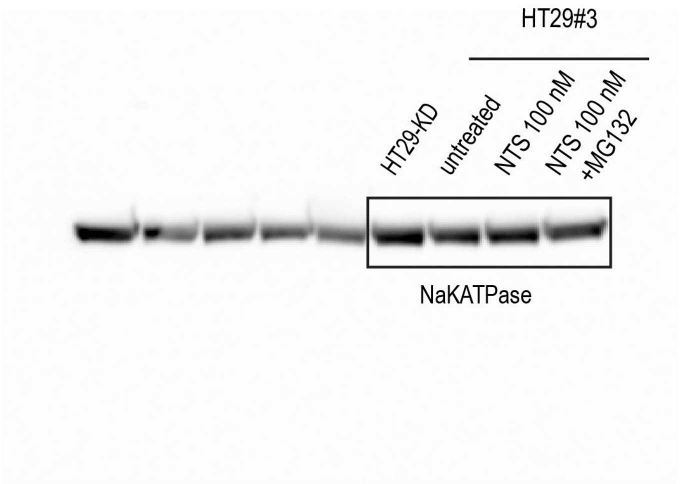

E

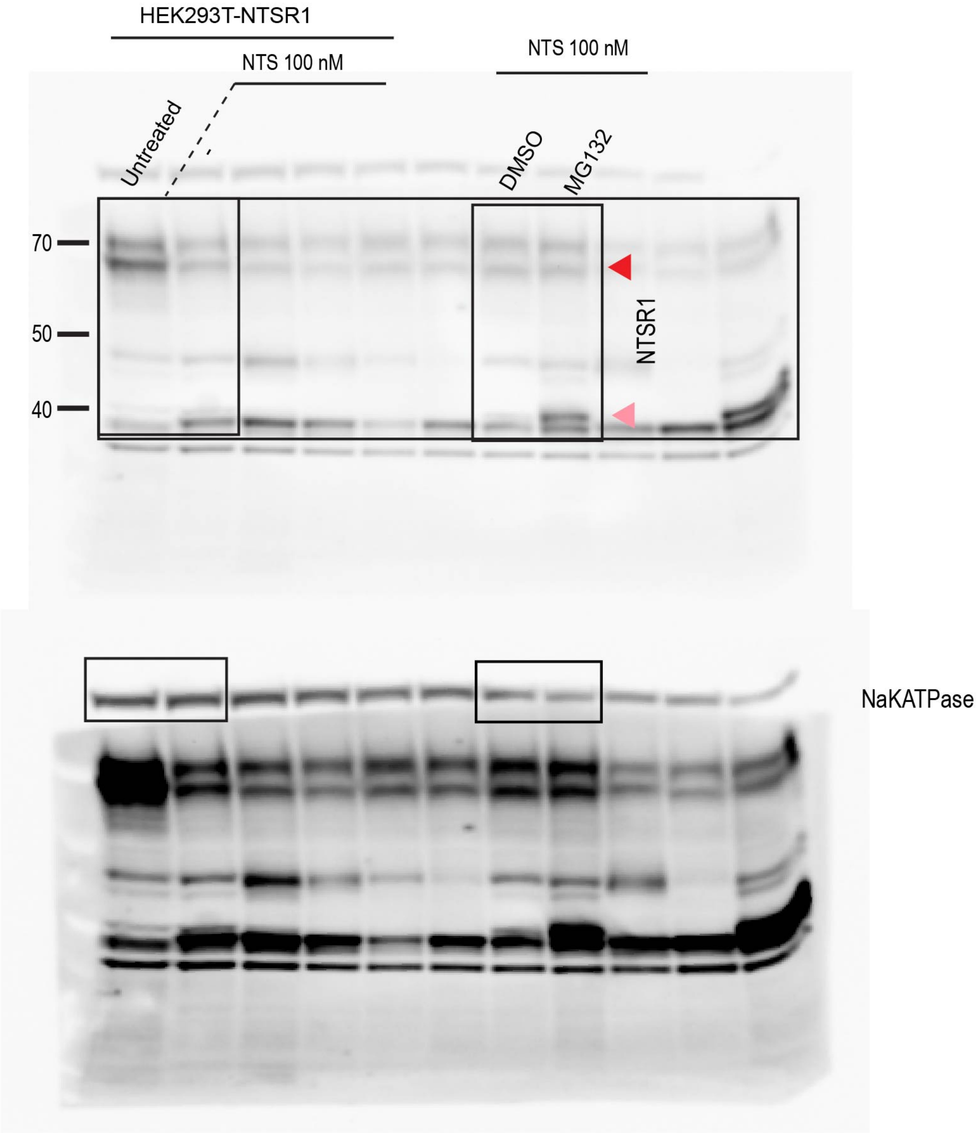

Supplementary figure 1: NTSR1 SDS/PAGE WB optimal conditions

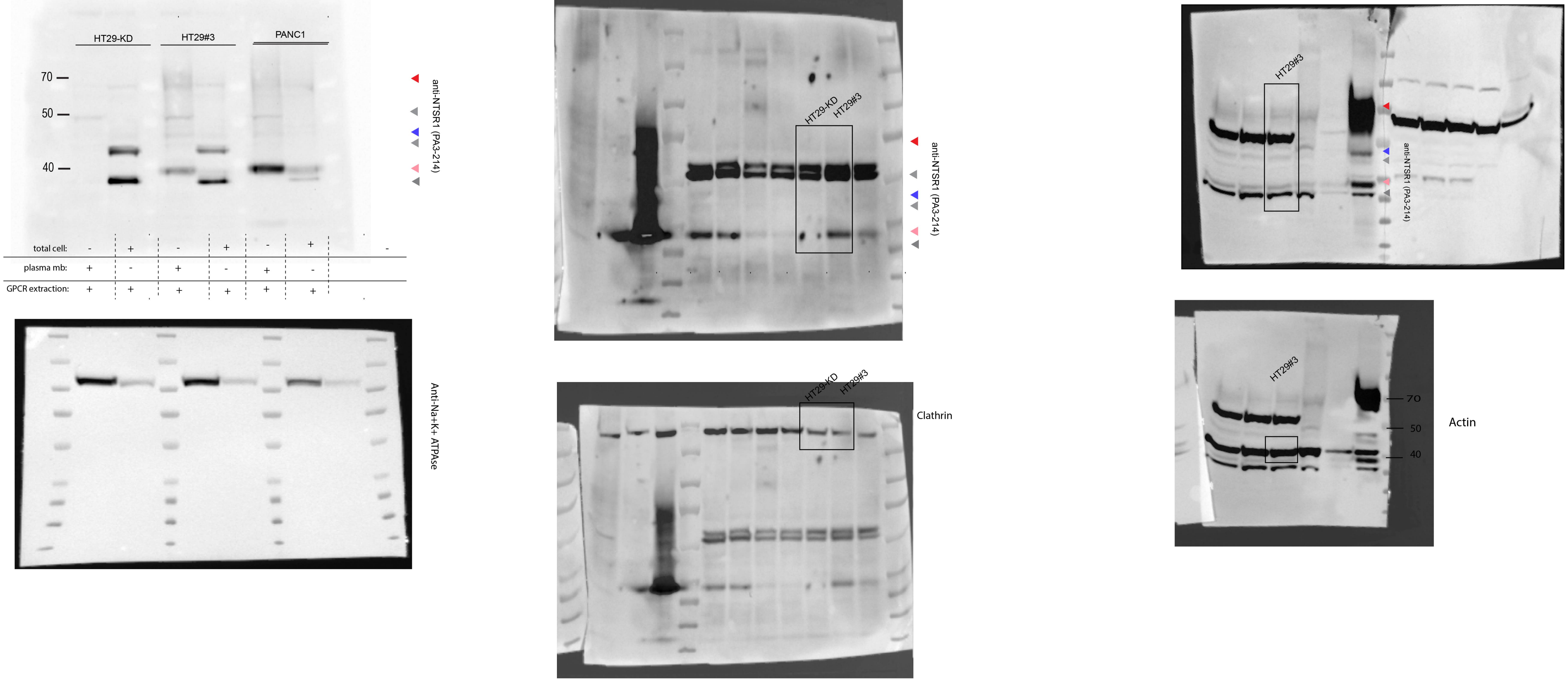

Supplementary figure 2 : NTSR1 protein depletion by siRNA, shRNA and CRISPR-cas9

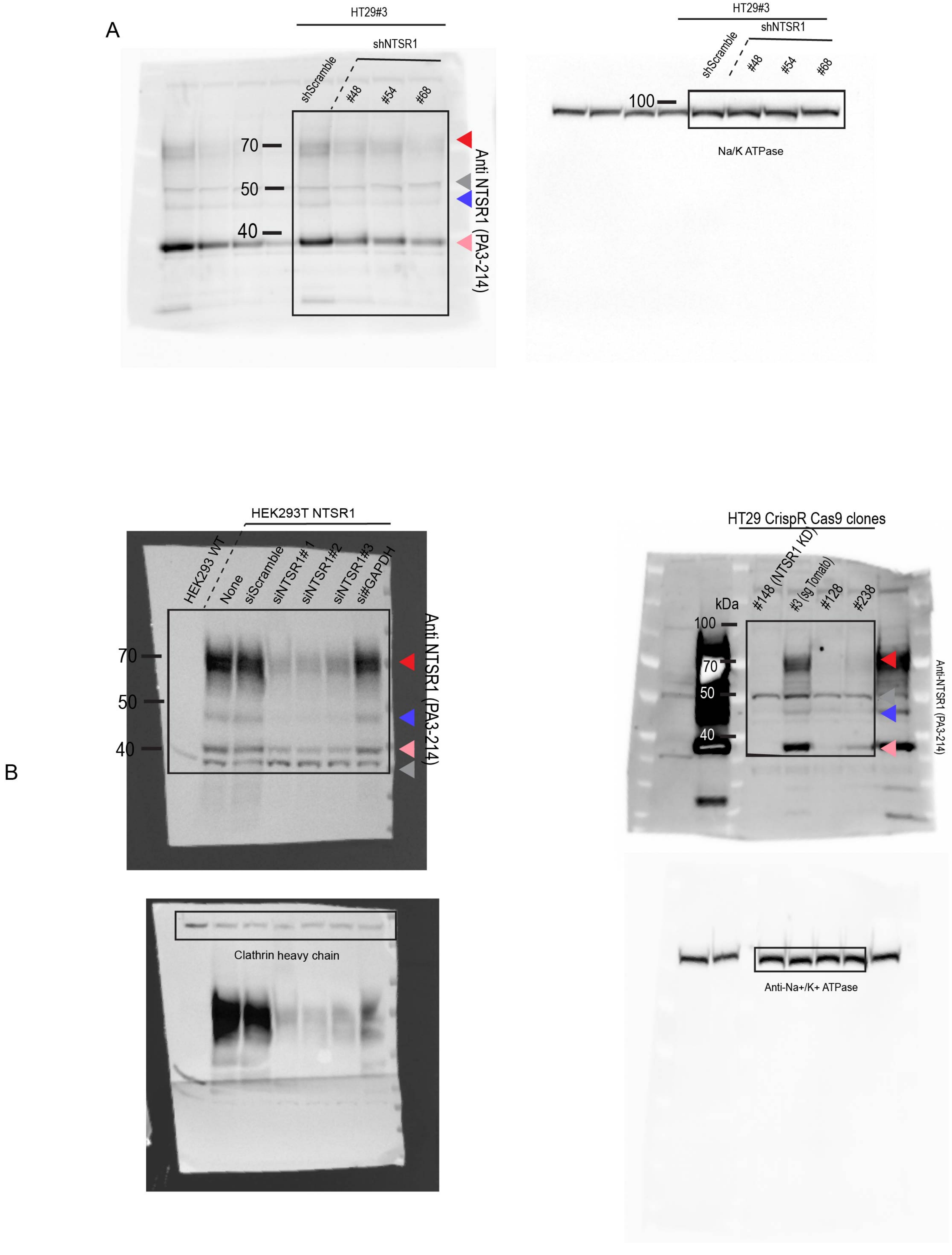

Supplementary figure 3 : NTSR1 protein construct

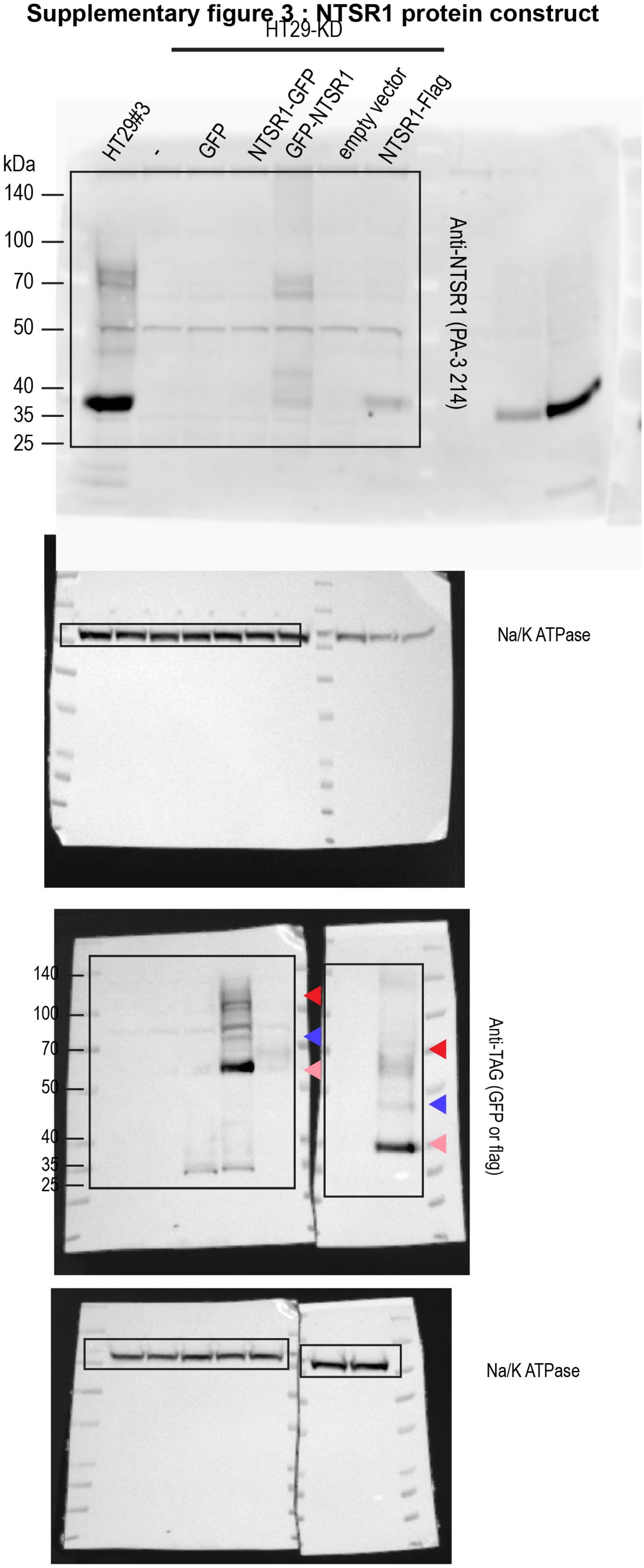

Supplementary figure 5 : NTSR1-high is not destabilized by denaturing agents

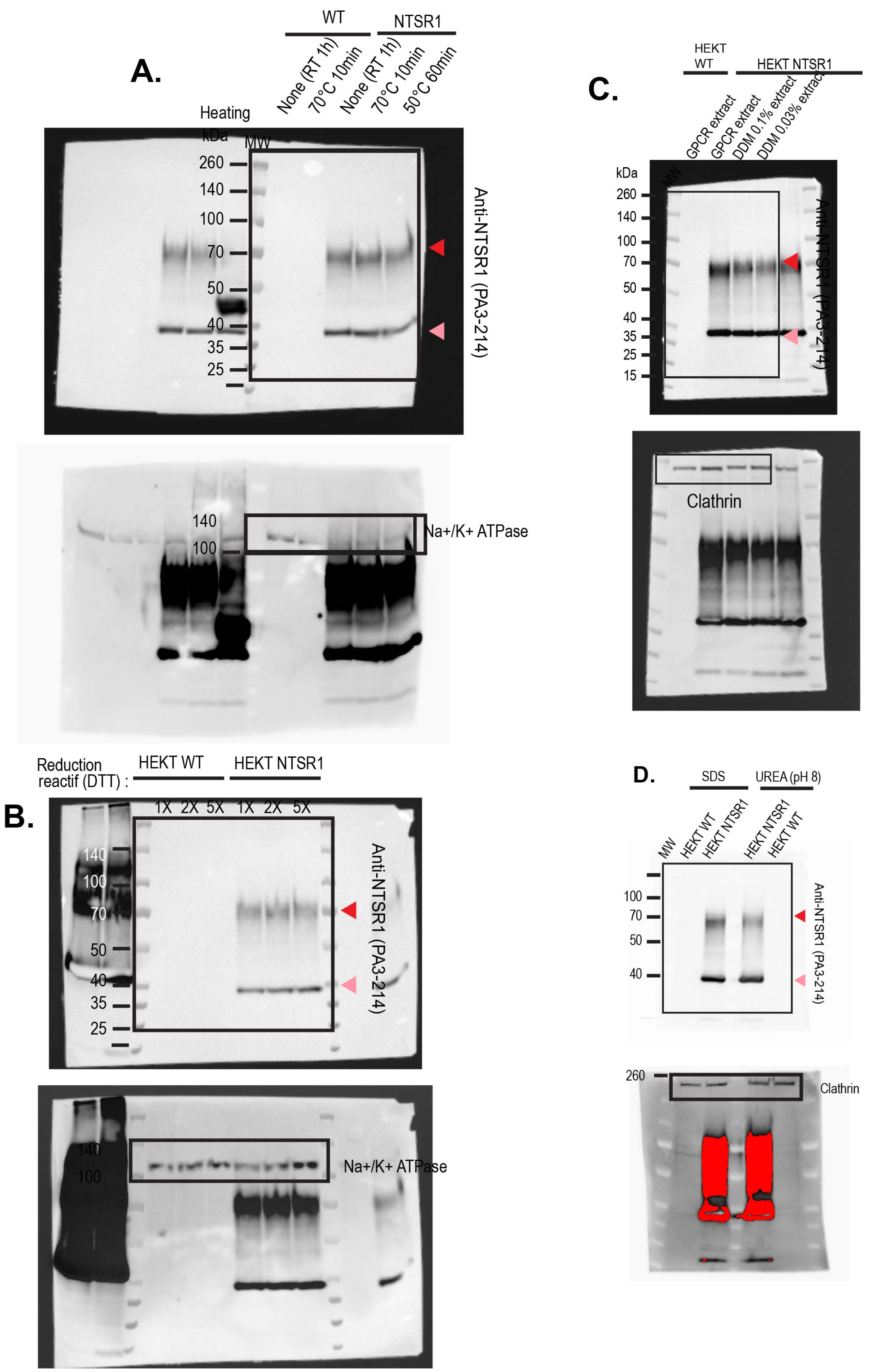

Supplementary figure 6 : NTSR1 is deglycosylated by PNGase F and BADG

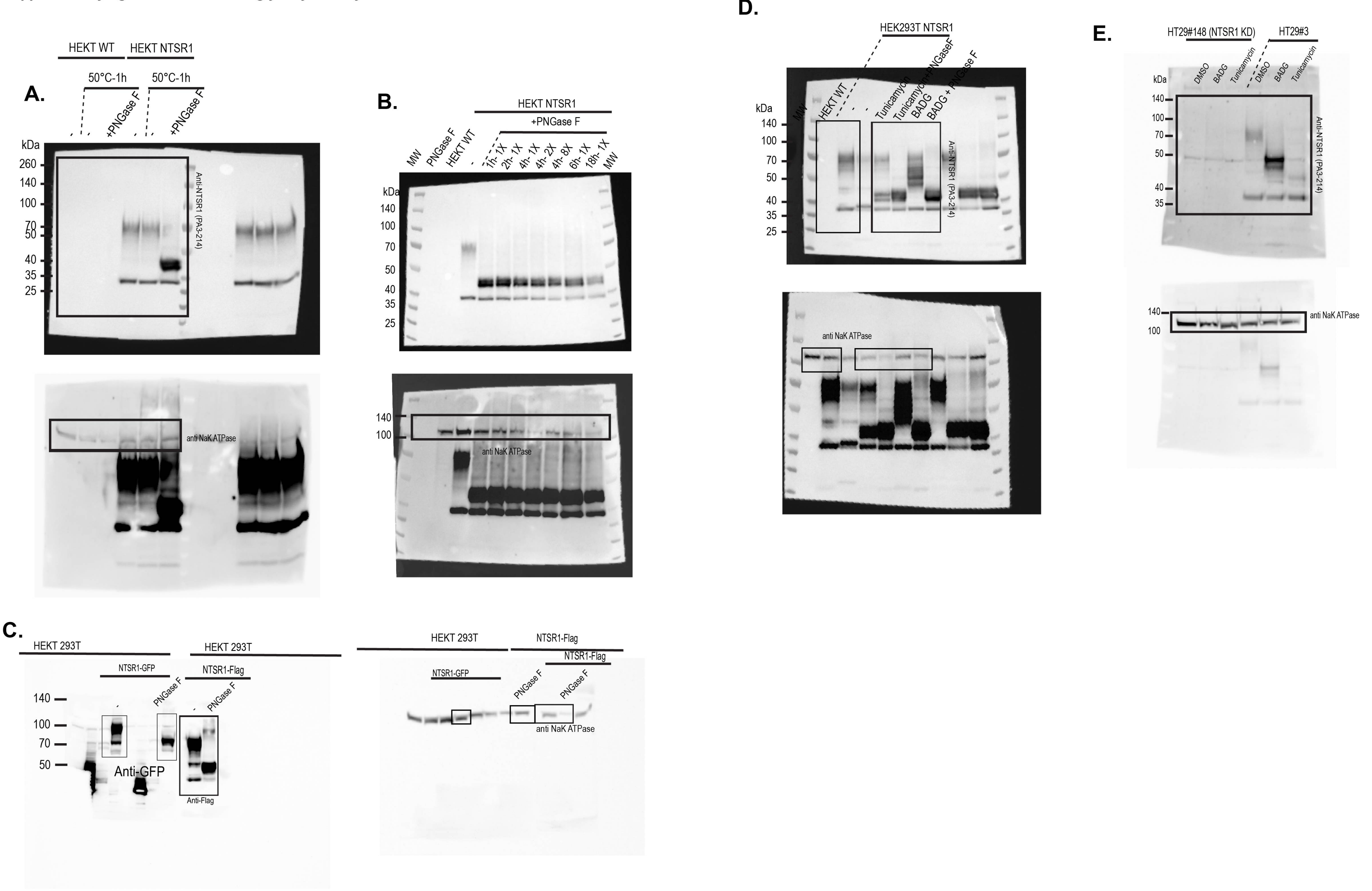

Supplementary figure 8 : NTSR1 expression in CRC PDX

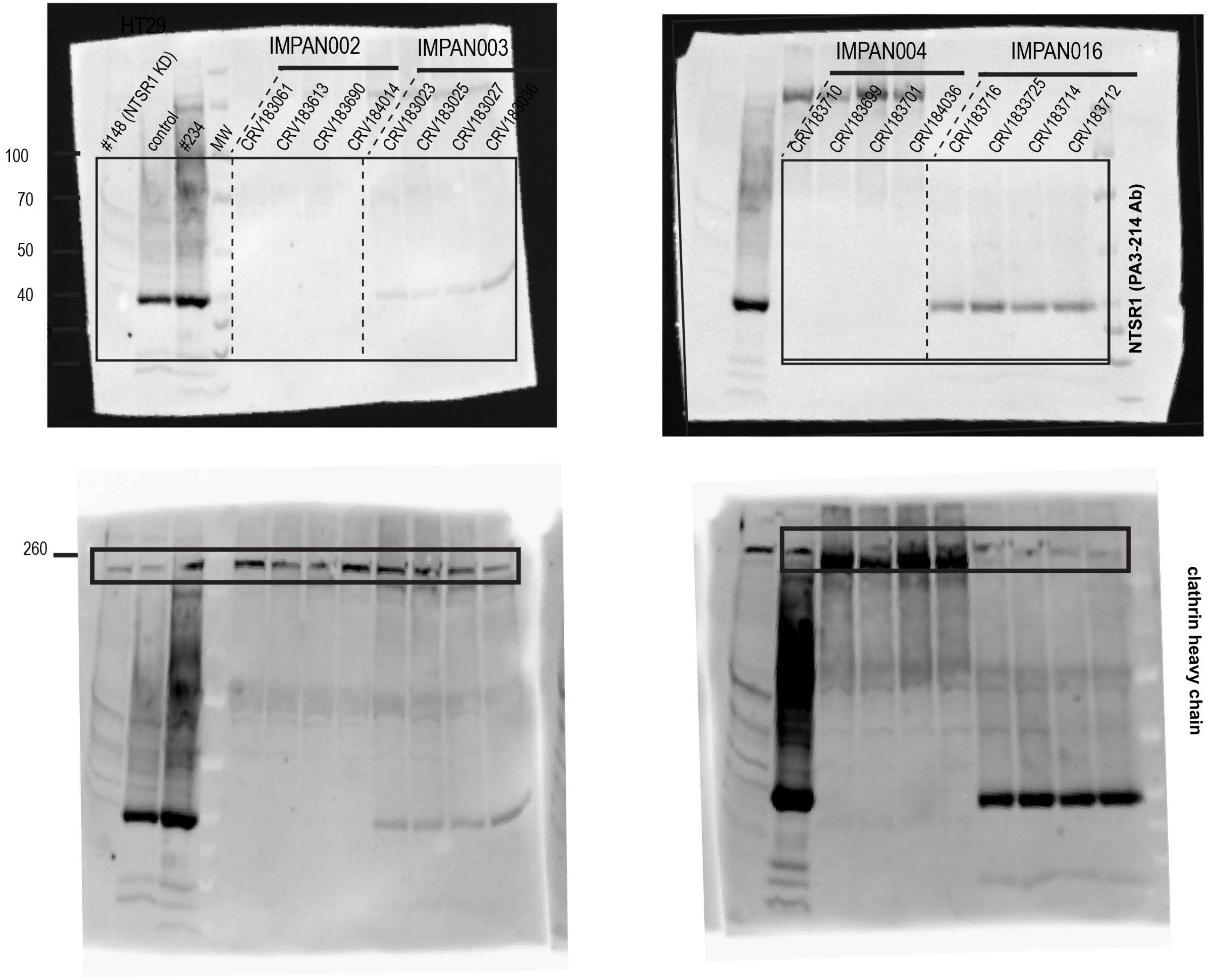

Supplementary Figure 9 : Lysosomal degradation is not involved in NTSR1-low form degradation

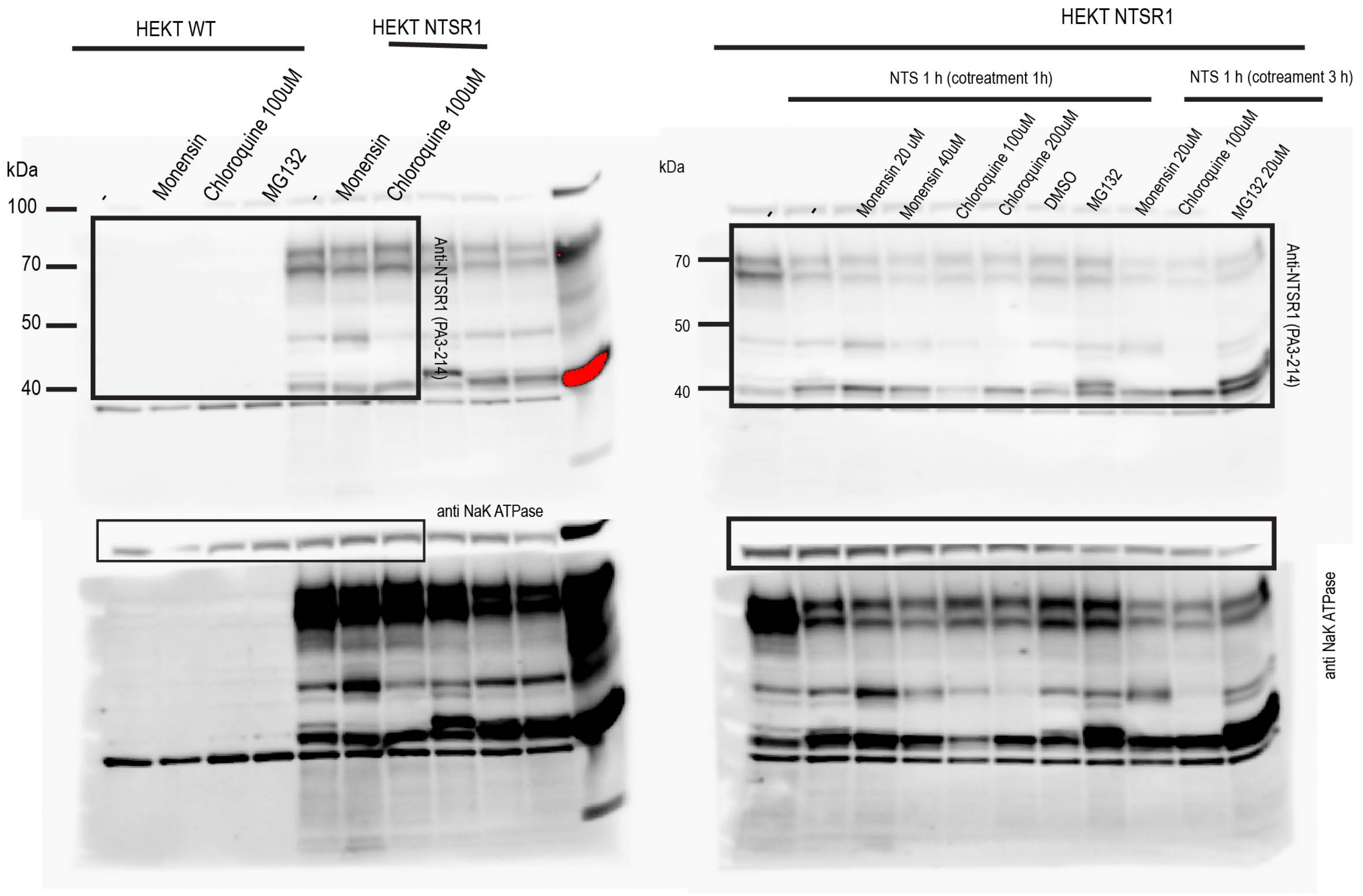

Supplement: Supplementary file 2 — Supplementary Information 2. [file 41598_2023_31790_MOESM2_ESM.pdf]
